# Novel﻿ markers to early detect degradation on cellulose nitrate-based heritage at the submicrometer level using synchrotron UV–VIS multispectral luminescence

**DOI:** 10.1038/s41598-021-99058-6

**Published:** 2021-10-12

**Authors:** Artur Neves, Ana Maria Ramos, Maria Elvira Callapez, Robert Friedel, Matthieu Réfrégiers, Mathieu Thoury, Maria João Melo

**Affiliations:** 1 LAQV/REQUIMTE and Department of Conservation and Restoration and Department of Chemistry, NOVA School of Science and Technology, Universidade NOVA de Lisboa, 2829-516 Caparica, Portugal; 2 Centro Interuniversitário de História das Ciências e da Tecnologia, Faculdade de Ciências, Universidade de Lisboa, Campo Grande, 1749-016 Lisbon, Portugal; 3 Department of History, University of Maryland, College Park, MD 20742 USA; 4 Synchrotron SOLEIL, l’Orme des Merisiers, St. Aubin, BP48, 91192 Gif-sur-Yvette, France; 5 Centre de Biophysique Moléculaire, CNRS UPR4301, Rue Charles Sadron, 45071 Orléans, France; 6 Université Paris-Saclay, CNRS, Ministère de la Culture, UVSQ, MHNH, IPANEMA, St. Aubin, BP48, 91192 Gif-sur-Yvette, France

**Keywords:** Optics and photonics, History of chemistry, Photochemistry, Physical chemistry

## Abstract

Cellulose nitrate (CN) is an intrinsically unstable material that puts at risk the preservation of a great variety of objects in heritage collections, also posing threats to human health. For this reason, a detailed investigation of its degradation mechanisms is necessary to develop sustainable conservation strategies. To investigate novel probes of degradation, we implemented deep UV photoluminescence micro spectral-imaging, for the first time, to characterize a corpus of historical systems composed of cellulose nitrate. The analysis of cinematographic films and everyday objects dated from the nineteenth c./early twentieth c. (Perlov's collection), as well as of photo-aged CN and celluloid references allowed the identification of novel markers that correlate with different stages of CN degradation in artworks, providing insight into the role played by plasticizers, fillers, and other additives in stability. By comparison with photoaged references of CN and celluloid (70% CN and 30% camphor), it was possible to correlate camphor concentration with a higher rate of degradation of the cinematographic films. Furthermore, the present study investigates, at the sub-microscale, materials heterogeneity that correlates to the artworks' history, associating the different emission profiles of zinc oxide to specific color formulations used in the late nineteenth and early twentieth centuries.

## Introduction

### A pioneer plastic in peril

**Figure 1:**
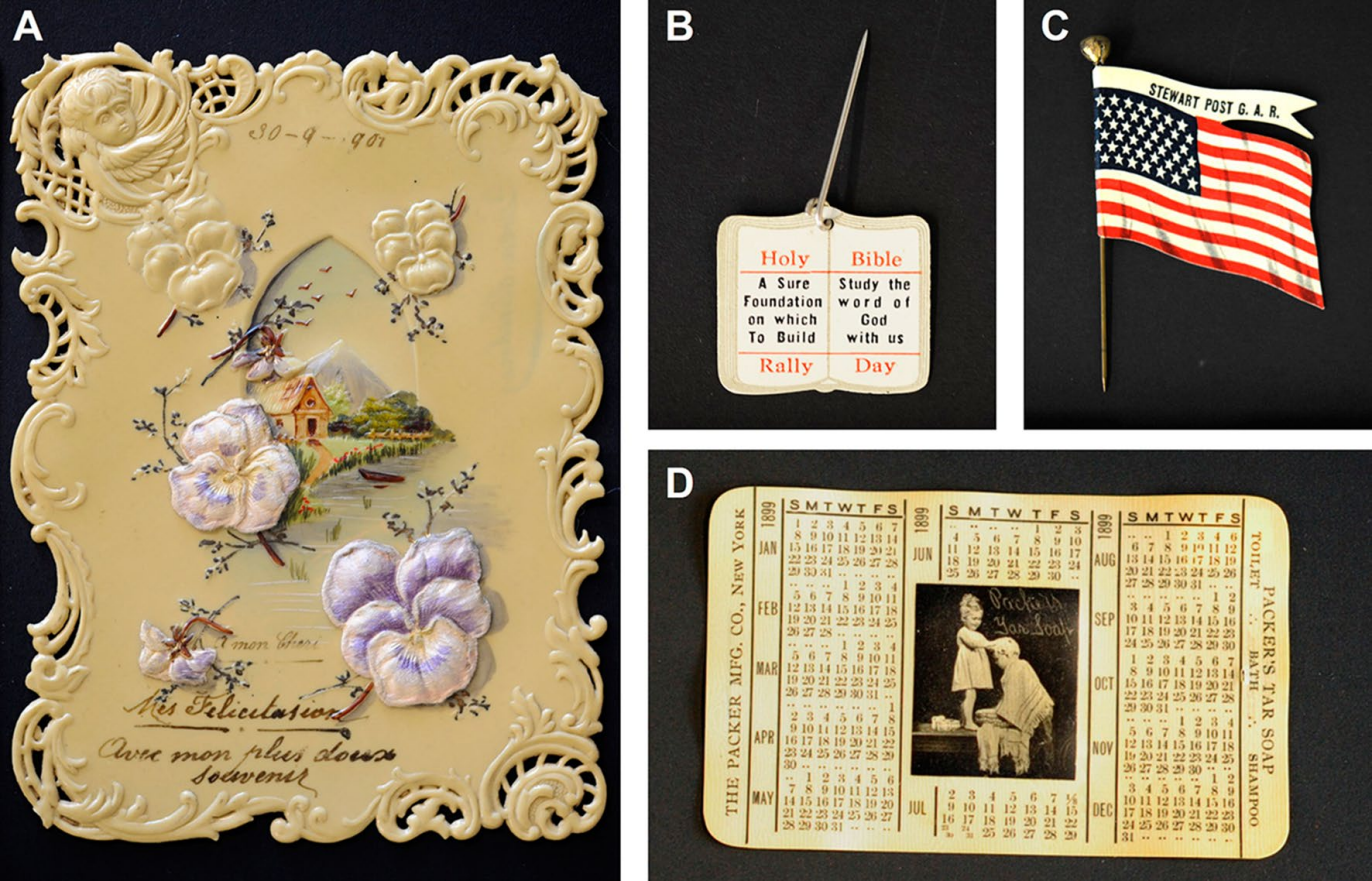
Objects from Perlov’s celluloid collection that consists of 300 everyday celluloid objects, donated by Amy Schenkein and Dadie Perlov, American collectors and majority donors of the Smithsonian Institute's celluloid collection. Containing several objects of different types, from razor blades, fans, pins, postcards or advertising items, this collection is significant of the American technological advances in the celluloid industry from the 1890s to the 50s. The objects showed were studied in the framework of this research: a 1901 postcard (**A**), two undated advertisement pins, hereby called the holy bible pin (**B**) and the American flag pin (**C**), and an 1899 calendar (**D**).

Cellulose nitrate is considered the first semisynthetic polymer and was trademarked as Parkesine and, later in the USA, as Celluloid by John Wesley Hyatt^1,2^. Its flexibility and dimensional stability led to its extensive use as a new photographic and cinematographic medium for films, being present in image heritage collections in archives and museums^3,4^. It was also widely used between the 1890s and 1920s for design pieces and everyday objects, all testimonies of our material culture, Figs. 1 and 2. Celluloid also attracted artists like Naum Gabo and Antoine Pevsner to creating sculptures that are now preserved in museums^5^. This heritage may be at risk, as we do not yet know what factors trigger an irreversible degradation leading to the total loss of these precious objects. It is for this reason that cellulose nitrate is considered an intrinsically unstable material in heritage collections, posing also risks to human health. To slow down degradation, low-temperature storage is accepted by the conservation community^6^, but these solutions prohibit public access, are price-sensitive, have high energy costs and there are concerns about its effects on the physical stability and material lifetime^7,8^. Important insights on its degradation were achieved over the last decade^7–32^, but a full elucidation of its mechanisms is still required to develop sustainable conservation strategies as in the NEMOSINE project, by developing a smart modular package with the main goal of energy-saving and extent conservation time^33–35^.

**﻿Figure 2:**
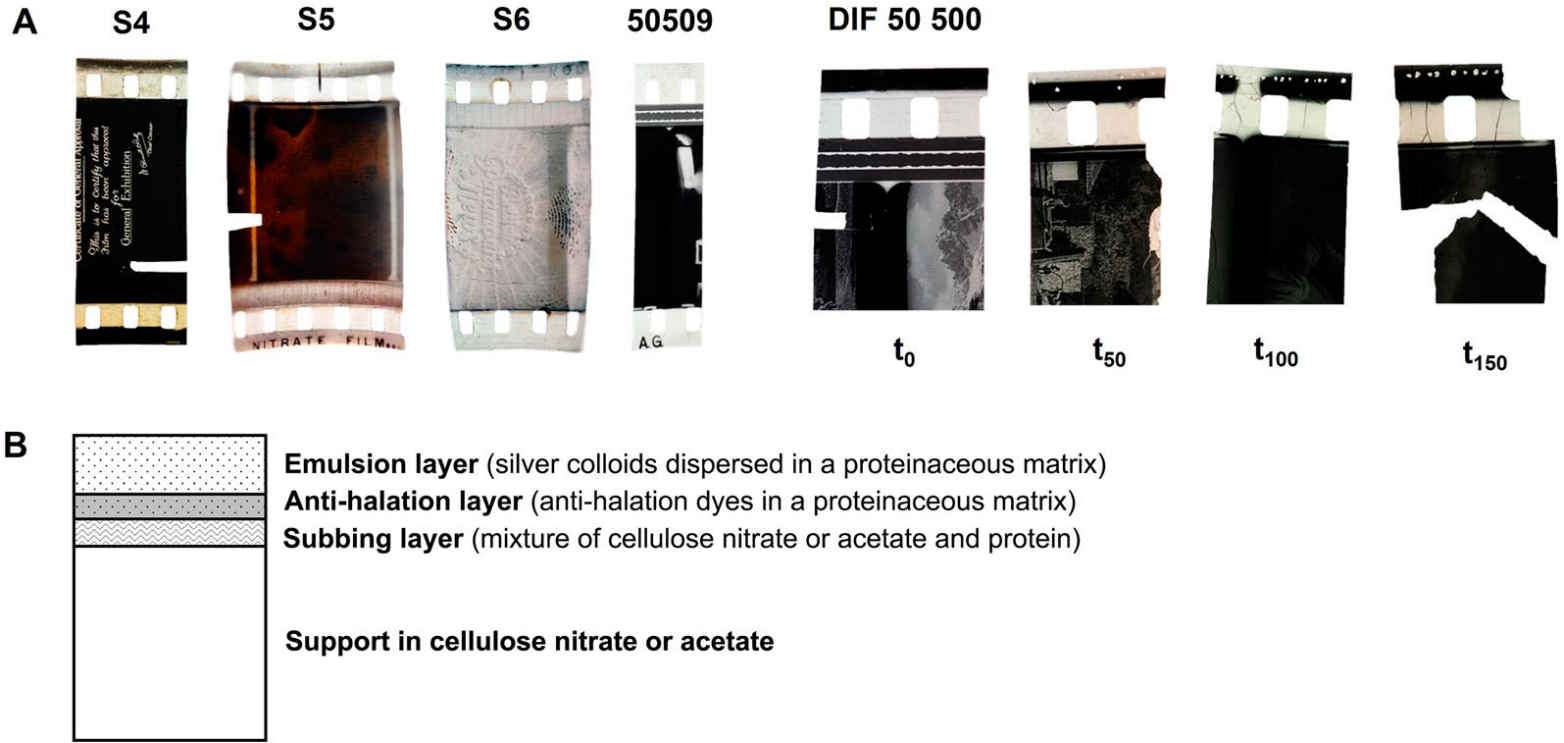
(**A**) Cinematographic film samples S4, S5, S6, 50 509 and DIF 50 500. DIF 50 500 was artificially aged (λ ≥ 280 nm, 40 °C): at 50 h of irradiation the film was yellowed and fragile; at 100 h presented cracking; and at 150 h the film lost its integrity. (**B**) Multi-layer structure of a generic cinematographic film. The cellulose nitrate is the thickest layer and it is the support of the image. The image is formed by silver colloids dispersed in a proteinaceous matrix which is adhered to the support by another thin layer, the subbing layer. Finally, the anti-halation layer prevents light to be reflected in the image layer causing a halo effect.

### Celluloid in private and public collections of plastics

During its heyday (roughly from 1880 to the 1920s) celluloid was used for an astonishing array of toiletries, novelties, and “fancy goods”. It was prized particularly for the ease with which it could be made to imitate semi-precious materials like ivory, tortoiseshell, coral, and mother-of-pearl^36^. Almost every article of commerce that could be made of these materials was fabricated from celluloid, and generally (but not always) sold more cheaply than they. For the most part, celluloid was not in any way functionally inferior to the materials it replaced, and it was frequently aesthetically equivalent^36^. In some forms, its cost was so low that it could be used in large volume for ephemeral articles—often advertising novelties (such as pocket calendars, rulers, book-marks, trade cards, and the like)^1^. Because these pieces were both more durable and more handsome than the card or paper items they replaced, they were often kept and stashed away rather than disposed of. Larger objects, such as combs, brushes, and other toiletry items, were often passed down as heirlooms, much as their ivory or tortoiseshell models would have been.

In the twentieth century, celluloid was almost everywhere eclipsed by newer and generally less expensive plastics. Even in photography and cinema, the material was replaced by the less flammable acetate films. In consumer goods, from toys to toiletries to tools, celluloid gave way to the products of chemical industry, led at first by phenol–formaldehyde (introduced as Bakelite) and then in even greater volume by a host of synthetic polymers, mostly made from petrochemicals. Celluloid became almost quaint as its uses fell away, until by mid-century not much more than table tennis balls and guitar picks remained^36^. But celluloid remains well represented in private and public collections of plastics, and the objects found in those collections raise a host of issues for understanding both the behavior of the material and the ways in which we use the material and the objects for historic preservation and representation.

The study here attempts to demonstrate methods for investigating the historical and physical experience of this first plastic in cinematographic films (dated from 1925 to 1960) and objects, Fig. 2 and Table 1. The objects are part of a collection that originated with the private efforts of Dadie Perlov, who collected a wide range of small celluloid articles, largely from the northeastern United States during the last decades of the twentieth century. Items from Perlov’s collection constitute the primary celluloid holding in the museums of the Smithsonian Institution, in Washington, D.C^36^. Other items have ended up in other museums, and about 304 have been donated to Portuguese institutions, and these have been the basis of the study here.

**Table 1:** Description and degree of substitution (DS) of the cinematographic films and celluloid objects studied (Perlov’s celluloid collection).

Samples	DS^a^	Thickness (µm)	Date	Manufacturer	Description
S4	2.15	193	1932–1942	Kodak	35 mm black and white cinematographic film with no sound
S5	2.20	187	1933–1942	Kodak	35 mm black and white cinematographic films with sound
S6	2.06	205	N.A	Agfa	
50 509	2.18	147	1930	Kodak	
DIF 50 500	1.71	175	1940–1960	Kodak	
Postcard	2.06	300	Possibly from 1901	N.A	Celluloid postcard with floral and landscape-colored reliefs. Handwritings in French. In the upper part of the postcard, the date “30–09-1901” is written
Holy Bible Pin	2.00	400	N.A	N.A	A celluloid pin with a metallic needle. On the back has the inscription “Made in the USA”
American Flag Pin	2.01	300	N.A	Bastian Bros, New York	A celluloid pin with a metallic needle. This pin belonged to the Grand Army of the Republic (G.A.R.)
Calendar	1.88	1000	1899	The Whitehead and Hoag Co., New Jersey	An 1899 celluloid year calendar with an advertisement to The Packer Manufacturing company in New York

^a^Degree of substitution calculated by µFTIR using the calibration curve described in Nunes et al.^33^.

### Synchrotron deep UV photoluminescence micro spectral-imaging to safeguard celluloid heritage

Celluloid nitrate absorbs and can be excited in the UV (Supplementary Fig. S1), emitting in the UV–VIS region^37^; early degradation products that are formed in extremely low concentrations are also prone to show specific luminescence signals, making photoluminescence techniques very adapted to identify chemical changes^38^. In this work, we propose to assess the level of degradation by the use of luminescent chemical markers that are excited in the deep UV (DUV) using two instruments operating between 200 and 800 nm. The first endstation is dedicated to raster micro-scanning photoluminescence spectroscopy using a confocal configuration (POLYPHEME)^39,40^. The second one is a multispectral imaging system based on a full-field configuration (TELEMOS)^39,40^. In this specific configuration, using synchrotron beam as a source of light to generate photoluminescence imaging, the limit of detection can be approached to 100 nM, enabling to detect intermediates and products that will be invisible to most spectroscopy techniques, due to their very low concentration^38^.

In addition, the possibility to retrieve centimetric fields of view at sub-micrometer lateral resolution has shown to be extremely efficient for tackling the intrinsic heterogeneity of the historical objects^39,40^, such as those studied in this work, which can be described as intrinsically heterogeneous systems, Table 1 and Figs. 1 and 2. Cinematographic films can be described as multilayer systems in which CN-based polymers are used as image support: pigments or fillers are not expected in this type of matrix. In contrast, in the celluloid artworks from the Perlov’s collection, fillers and pigments are present, admixed with celluloid. The analysis of these complex historical samples is supported by highly characterized reference materials, artificially aged, which will be studied both using DUV micro-imaging and UV–VIS spectrofluorimetry. Historical films were also artificially aged to simulate longer periods of natural ageing.

**Figure 3:**
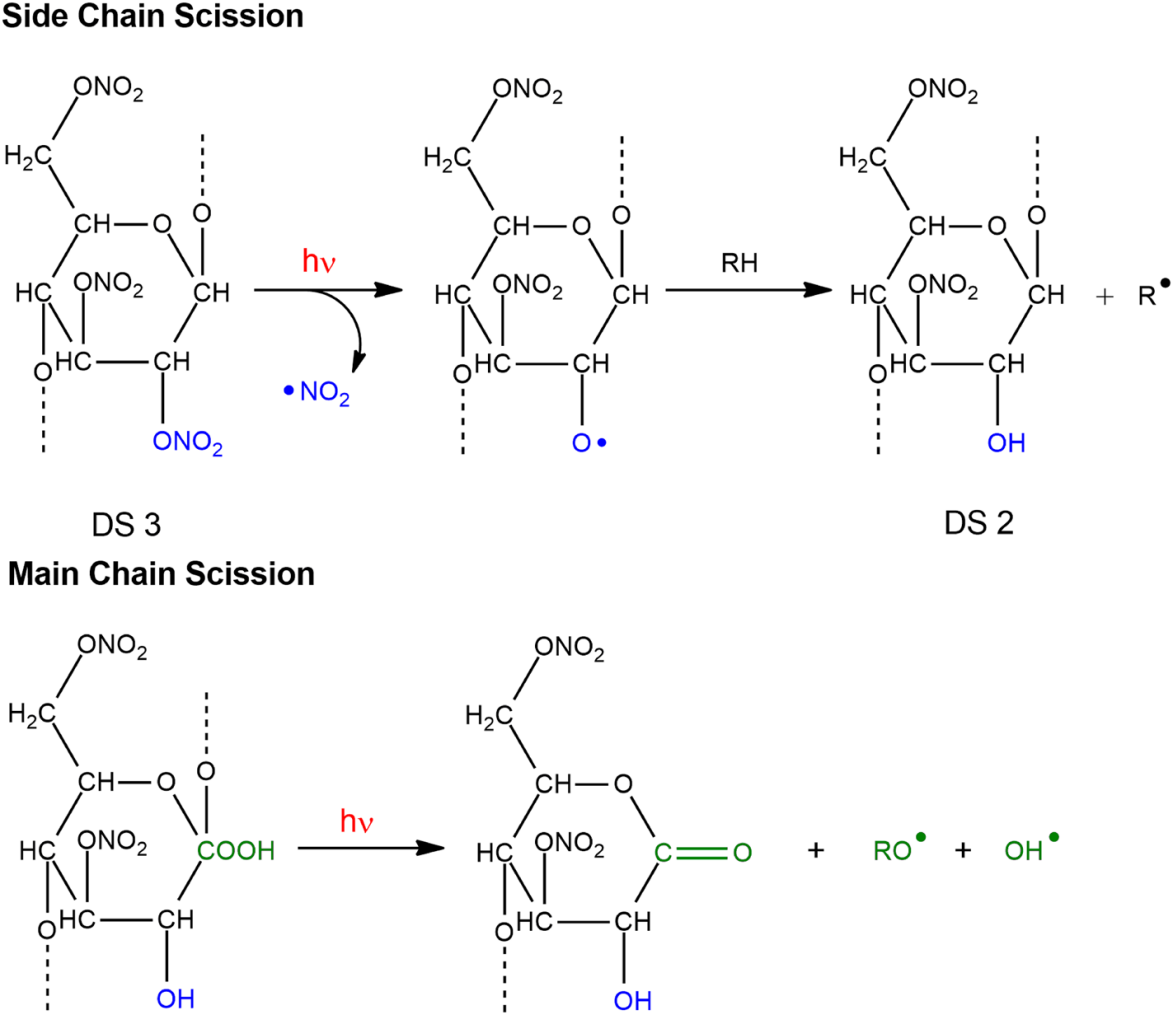
Cellulose nitrate side-chain scission starts with de-nitration, by the homolysis of a nitrate group in C2 or C3 and the release of •NO_2_ (inducing a DS decrease). In a second phase, hydroperoxides formed in C1, lead to main chain scission through cleavage of the glycosidic bond, with the formation of a gluconolactone as the primary degradation product. This mechanism, triggered by light, produce excited states that can simulate the natural ageing.

In this work, the level of degradation assessed by the luminescent markers is compared with the degree of substitution (DS) of CN calculated by infrared spectroscopy, since DS decreases with ageing^20,26,28,29,33,41^. This decrease in DS in CN-based polymers, is a consequence of the first phase of the degradation mechanism, which occurs through homolytic scission of the nitrate groups^19,26^. In a pristine matrix used as support for cinematographic films, the DS is expected to be ca 2.26^33,42^, and its decrease is a consequence of the scission of the nitrate group and its substitution by hydroxyl groups as depicted in Fig. 3. In the first phase of degradation, this leads to an increase and broadening of the carbonyl band at 1740 cm^−1^ alongside the increase of the OH absorption between 3700 and 3100 cm^−1^, in the infrared spectrum^26,32,33^. For more details, please see next section.

The data acquired by synchrotron deep UV photoluminescence micro spectral-imaging (DUV-µPL), will be for the first time used to identify alteration markers in historical objects made from CN. This knowledge can support the development of early warning tools to monitor CN degradation in heritage collections.

### Photooxidation of cellulose nitrate to elucidate its degradation mechanism

Recent insights into the degradation mechanism of cellulose nitrate and celluloid were achieved by Therias and Gardette et al. These authors showed that CN and celluloid present a common degradation mechanism, being proved that photodegradation (*λ*_irr_ > 300 nm) simulates the natural ageing mechanism^19,26,32^. In a first phase, for CN, the results show that the main degradation mechanism is based on de-nitration, followed by the formation of oxidation products exhibiting lactone and anhydride functions detected by their infrared carbonyl band at 1735–1740 cm^−1^ and 1760 cm^−1^, respectively. Gluconolactone is identified “as the primary degradation product”, Fig. 3. In a second phase, these oxidized chemical structures will react, provoking side and main chain scissions, leading to physical modifications, which include embrittlement and yellowing^26,32^. In addition, Therias et al. correlated changes in the molecular weight distribution with the “loss of the pyranose and acetal structures and the formation of the gluconolactone”, by calculating the average number of chain scissions per chain^26^. Based on this research, an integrated version of the degradation mechanism of CN is presented in Supplementary Fig. S2, which is summarized in Fig. 3.

In our ageing experiments, irradiation is carried out at *λ*_irr_ > 280 nm because it simulates the mechanism observed by irradiating at *λ*_irr_ > 300 nm.

## Results and discussion

### Alteration markers in reference samples

**Figure 4:**
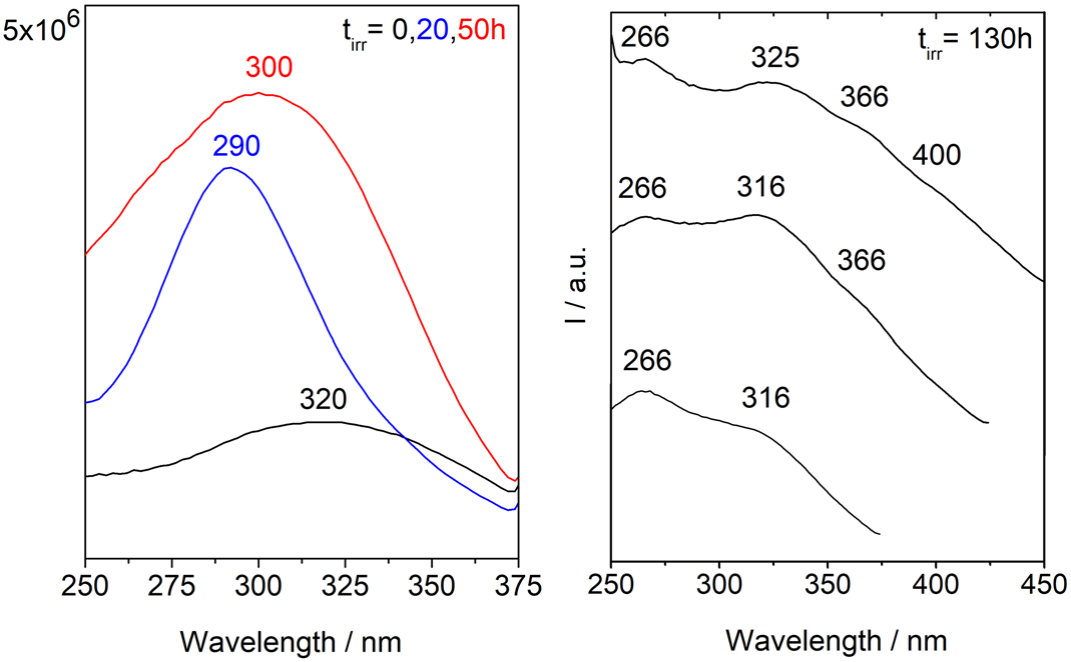
Irradiation of cellulose nitrate films followed by luminescence. Left, unreacted CN film is characterized by a broad excitation spectrum centred at 320 nm (λ_em_ = 390 nm), and in the first 20 h of irradiation the maximum is shifted to shorter wavelengths (290 nm) and then evolve to a maximum at circa 300 nm at 50 h of irradiation, which will continue to shift to longer wavelengths. Right, excitation spectra at 130 h of irradiation several oxidized functions are observed described by maxima at 266, 325, 366 and 400 nm (λ_em_ = 390, 450 and 480 nm). This agrees with the yellowing observed in the film.

**Figure 5:**
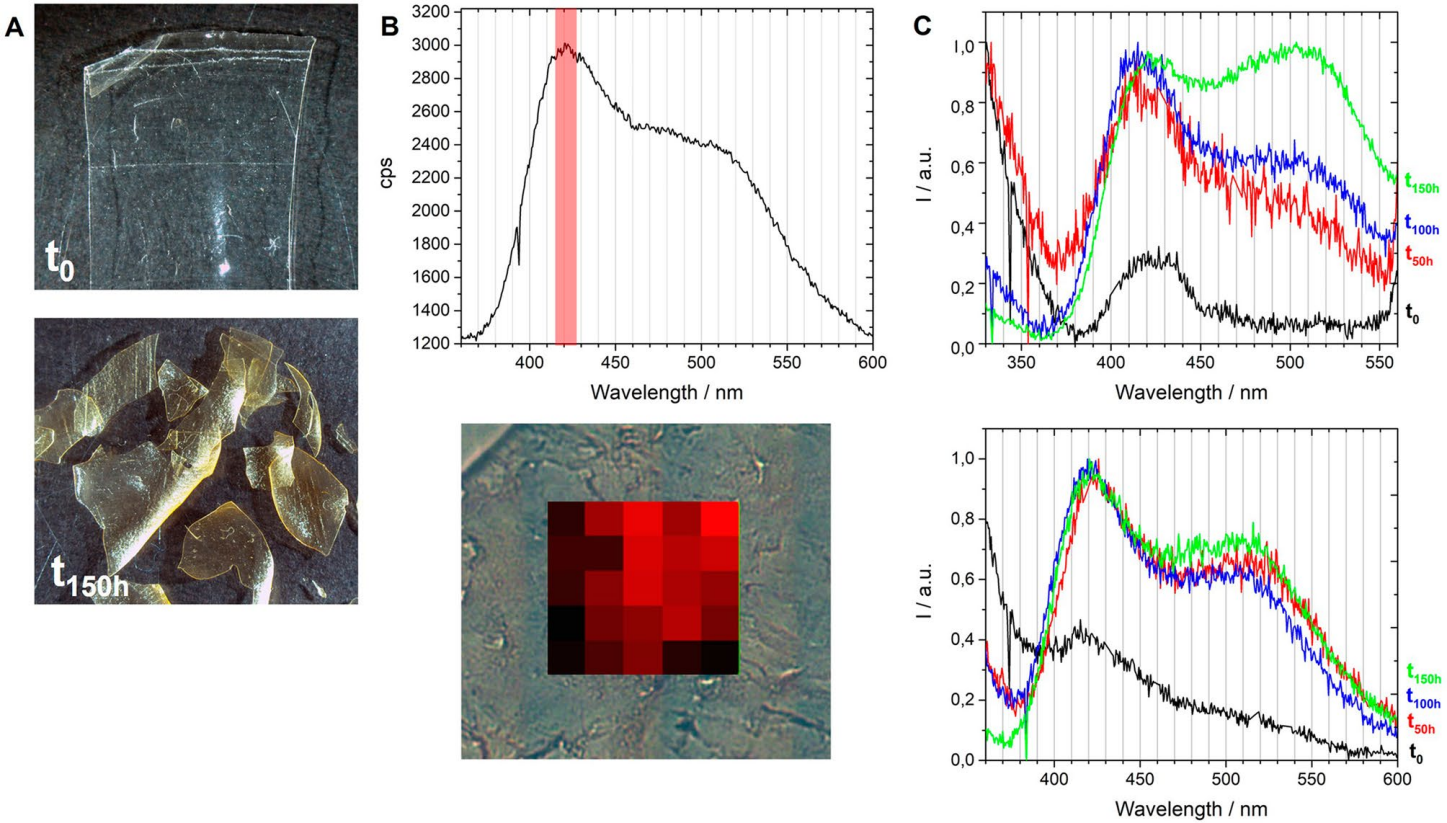
(**A**) Microscope images (7.1 × magnification) of cellulose nitrate homogeneous thin films (150 µm) used as reference films before (t_0_) and after 150 h of irradiation. (**B**) POLYPHEME raster scanning map of a 150 h aged celluloid reference (λ_exc_ = 330 nm, 20 µm step, 5 s acquisition time), together with the average, representative, emission spectrum. Intensity variations of the probed region between 415 and 425 nm are due to the topography of the sample. (**C**) Normalized emission spectra of artificially aged cellulose nitrate (top, λ_exc_ = 290 nm) and celluloid (bottom, λ_exc_ = 330 nm) irradiated during 50 h, 100 h and 150 h.

Fluorescence emission and excitation spectra collected on artificially aged reference samples show that different stages of CN degradation correlate with the detection of luminescent intermediates that display specific spectra, Fig. 4. A fluorescence excitation spectrum mimics the UV–VIS absorption spectrum of the chromophore that emits, and these were acquired using spectrofluorimetry (spot analyses, 10 × 2 mm^2^ using a Jobin Yvon spectrofluorometer). Originally, an unreacted CN film is characterized by a broad excitation spectrum centered at 320 nm, which during the first 20 h of irradiation shifts to shorter wavelengths (290 nm) and only then it will evolve continuously to longer wavelengths, starting at 50 h of irradiation with a maximum at circa 300 nm, Fig. 4 (left). The first shift to 290 nm can result from the degradation of chromophores formed in the dark. In the films that have suffered extensive degradation, a strong shift to higher wavelengths is observed in the excitation spectra; for example, at 130 h of irradiation, several oxidized functions are identified by the presence of excitation bands whose maxima are at 266, 325, 366 and 400 nm Fig. 4 (right). This agrees with the yellowing observed in the cellulose nitrate films^32^. Based on the excitation spectra collected on the various samples, we selected the excitation wavelength of 290 nm to acquire maps of emission spectra with the DUV micro-imaging set-up of the DISCO beamline, Fig. 5. The overall signal collected at the microscale is consistent with the averaged signal collected using spectrofluorimetry, Supplementary Fig. S3. With an important advantage, the emission spectra obtained having a better resolution, it was possible to discriminate two bands in aged samples, in what was seen by spectrofluorimetry as an unresolved broad band, Supplementary Fig. S3. For this reason, we will only discuss the emission spectra acquired using synchrotron radiation. An important first result was to prove that degradation is homogeneous in the reference films, which was achieved by showing that the average spectrum obtained with POLYPHEME mapping is representative of the luminescence of each pixel, Fig. 5B. POLYPHEME shows that CN at t0 is characterized by a weak emission at 425 nm, this band increases during irradiation (*I*_max_ *ca.* 2600 cps). Also, a second band is formed and its intensity increases over time in comparison to the first band at 420 nm, being visible at 150 h of irradiation with maximum at 510–520 nm. This is an important result that was tested to assess the conservation condition of CN by plotting this ratio I_425nm_/I_510nm_ versus irradiation time. We observed a linear correlation between those two parameters showing that quantification of the degradation state of CN can be assessed through the ratio between these two bands (I_425nm_/I_510nm_), Supplementary Fig. S4: the higher the intensity of the CN2 band at 510–520 nm over the first band CN1 (*ca.* 420 nm), the higher the extent of degradation when compared with the DS values obtained by infrared spectroscopy. In future work, more irradiation times will be measured (shorter and longer) for CN and celluloid, to assess whether different mechanisms are at play.

When exciting at 290 nm, the spectrum of celluloid is dominated by the emission of camphor (*I*_max_ *ca.* 1900 cps). To avoid it and to collect only the CN emission, excitation was performed at 330 nm for celluloid references. As observed for CN, degraded celluloid is also characterized by a first band at 420 nm and a second one at 510–520 nm, Fig. 5C. However, the I_425nm_/I_510nm_ ratios indicate that comparing to CN, celluloid has degraded more rapidly: celluloid value of 3.25 at 50 h of irradiation is similar to CN at 100 h of irradiation, 3.07.

In summary, the studies on reference samples identified relevant markers to probe alteration in CN films. To assess the relevance of those markers in naturally aged samples, we performed similar analytical procedures in the cinematographic films and celluloid artworks.

### Cinematographic films dated from 1925 to 1960, naturally and artificially aged

**Figure 6:**
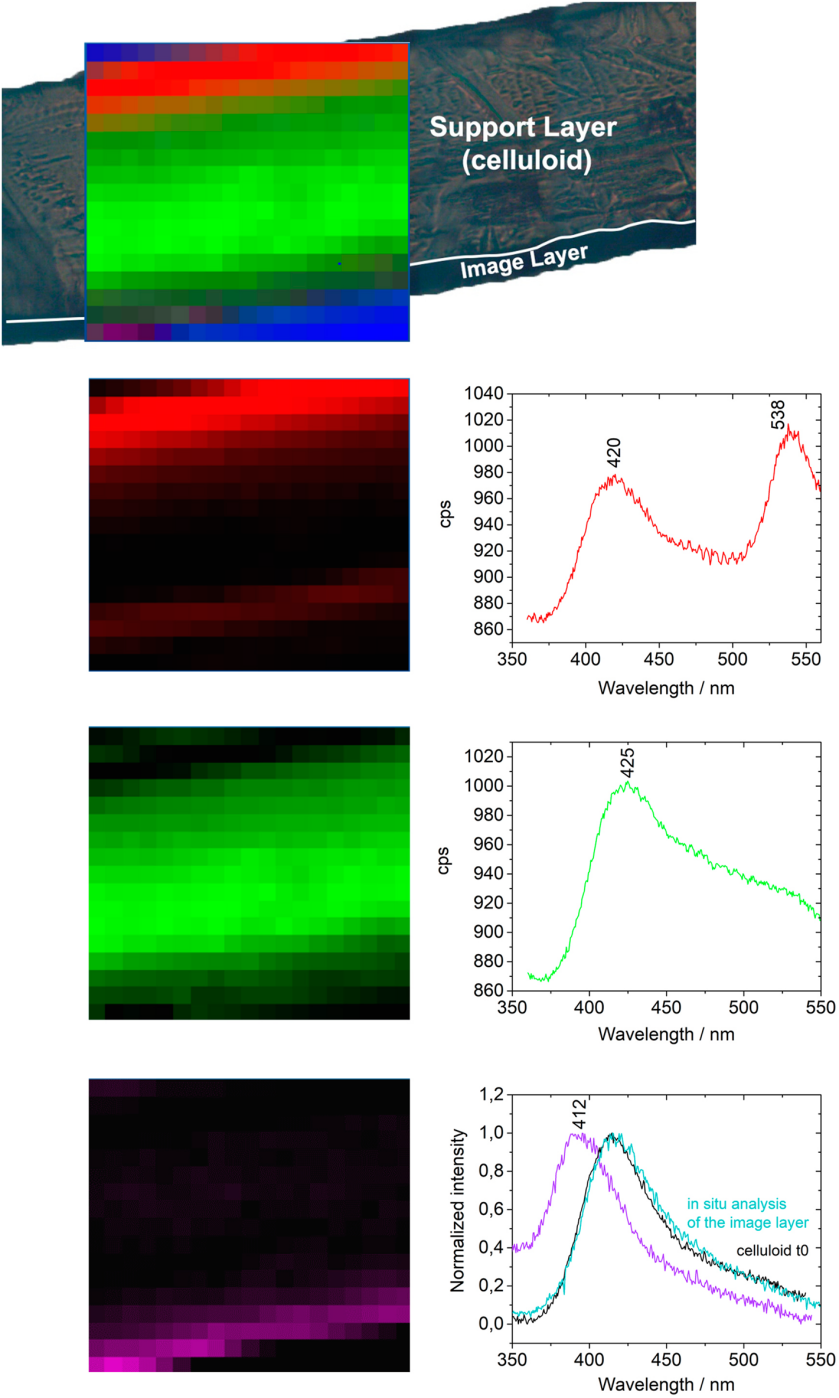
POLYPHEME raster scanning map (10 mm step, 5 s acquisition time, λ_exc_ = 330 nm) of the cinematographic film DIF 50 500. Three regions of interest were distinguished based on characteristic emission spectra: the proteinaceous image layer, with a band at 412 nm; the support layer with a band at 425 nm and a shoulder at higher wavelengths; and other products at the interfaces, air-support, and support-image, characterized by a band at 538 nm.

The observation of the stratigraphy of the cinematographic films studied shows that they are composed of the celluloid support and the image layer, Fig. 2B, which is a proteinaceous matrix with dispersed silver particles^43^. On naturally aged DIF-50500 cross-section, the spatial distribution of luminescent species collected using POLYPHEME allows to distinguish three regions by their specific emission spectra, Fig. 6: (1) in the proteinaceous material, the image layer was characterized by a band at 412 nm. This emission can be associated with collagen as it was made from animal sources, principally calfskin^44^; (2) in the celluloid support, the emission spectra presented a band at 425 nm and a shoulder at higher wavelengths (*ca.* 510 nm) that compare well with the 150 h irradiated celluloid reference (Fig. 5C) and, agree with the degree of substitution of 1.71 calculated for this film by infrared spectroscopy (indicative of a degraded CN)^33^, Table 1; (3) at the support interfaces, with air and with the image layer, the spectra presented two bands at 420 nm and 538 nm; this second band is more shifted and intense than the second band produced during degradation of celluloid references (centered at ca 510–520 nm), and can represent other types of degradation products. This emission at 538 nm can be related to a more extensive degradation or a different mechanism, Fig. 6. This degradation trend was confirmed in the cinematographic films S4, S5, S6 and 50509, where a more intense emission in the region between 500 and 550 nm at the interfaces was also observed, Supplementary Fig. S5.

DIF 50 500 samples artificially aged (*λ* ≥ 280 nm, 40 °C, 150 h) were analyzed in situ since it was not possible to prepare a cross-section due to the film brittleness, Fig. 2. After 150 h of irradiation, the emission spectrum acquired was very similar to that of the 150 h artificial aged cellulose nitrate reference emission, Supplementary Fig. S6. This behavior comparable to cellulose nitrate is probably due to the low amount of camphor in DIF 50 500, ca. 6% w/w, estimated by infrared spectroscopy^26^, Supplementary Fig. S7. This further strengthens the results obtained in the reference samples, proving that the higher the camphor concentration the higher the degradation rate  of celluloid.

### Nineteenth c./early twentieth c. celluloid everyday objects collection

Zinc oxide (ZnO) was the main pigment used to produce the ivory-like color and it was identified by its emission at 380 nm, in all the objects studied^39,40,45–47^. Zinc oxide is a semiconductor having a first narrow UV emission band at *ca.* 380 nm, assigned to the band-gap transition, and “its intensity represents a direct measure of the crystal quality”^48^. One or more emission bands in the visible region that tend to be broad and centered at ca. 425 nm and 510 nm^40,46,48^ can be attributed to defects or impurities that are related to levels located within the bandgap (trapped states). The ratio between the UV and visible bands can vary enormously in historical paints and may be used to distinguish between them^40,46^, Supplementary Fig. S8.

**Figure 7:**
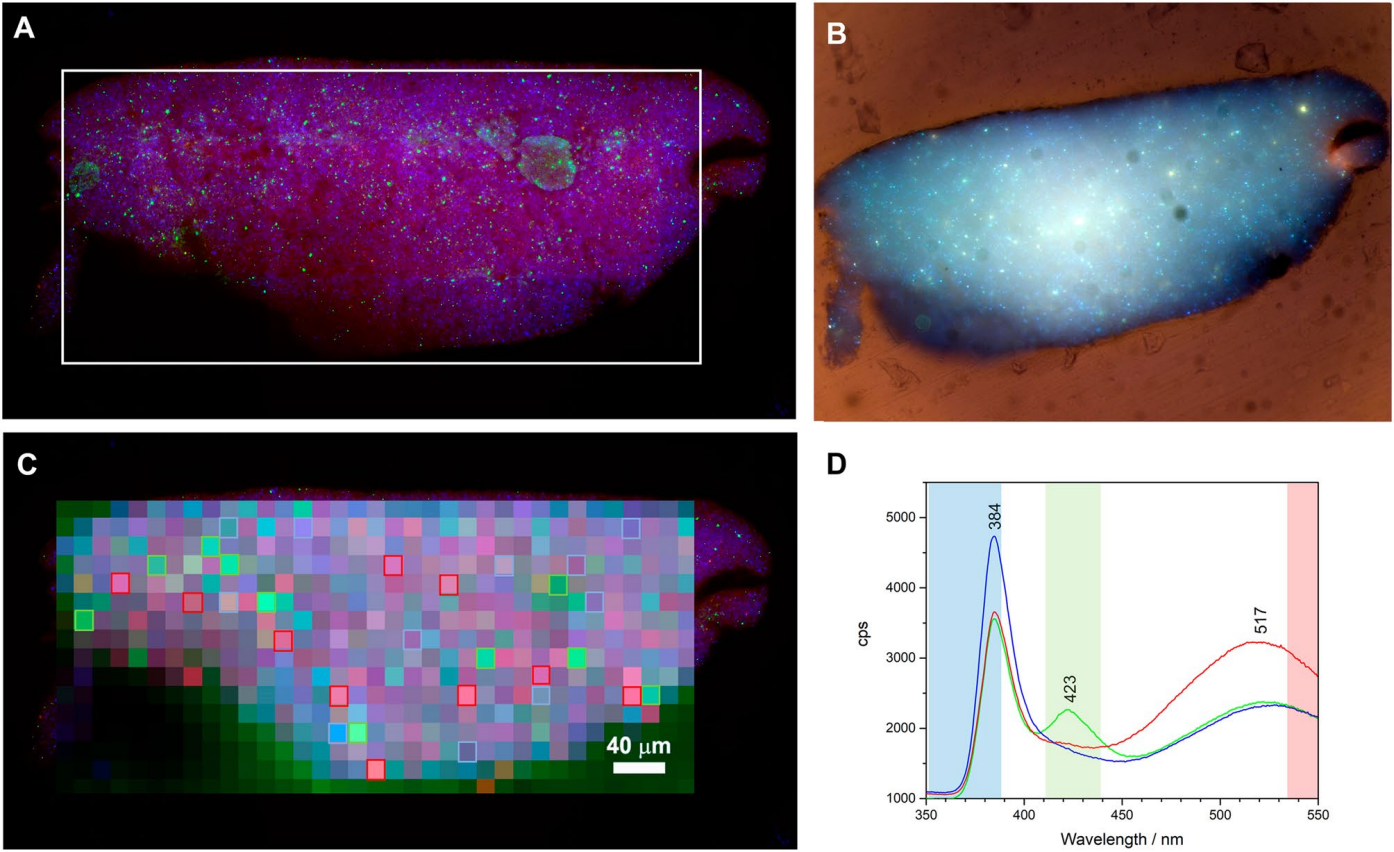
(**A**) Full-field luminescence imaging by TELEMOS of the American flag celluloid advertisement pin (λ_exc_ = 290 nm, 40 × objective). Emission bandpass filters used: 352–388 nm (blue); 412–438 nm (green); 535–607 nm (red). The white-square marks indicate the POLYPHEME map area. (**B**) Spatially registered false-colour RGB image of the emission at excitations of 365 (blue), 385 (green) and 405 nm (red) with emission bandpass filter 514 nm (30 nm FWHM). (**C**) Raster scanning map by POLYPHEME (15 × 15 µm^2^, 5 s, 2 accumulations, λ_exc_ = 290 nm). Colors were designated as follows: blue for the band edge emission at 385 nm, green for the spectral region between 400 and 450 nm; red for the region between 510 and 550 nm. (**D**) Average spectra calculated from 10 points for each designated color: strong near band edge emission (blue); contributions of ZnO crystal defect emissions between 400 and 450 nm (green) and strong green band emission (red). The spectral points used are marked in the POLYPHEME map (**C**) with squares of the correspondent color. The spectral regions viewed using TELEMOS setup are highlighted, with colours corresponding to the bandpass filters used.

In the case of the celluloid objects of the Perlov collection, following excitation at 290 nm, the three bands described for ZnO were observed, Fig. 7 and Supplementary Figs. S8–S14. These bands were identified with maxima at ca 380 nm (ZnO1), at ca. 418 nm (ZnO2) and at ca. 507 nm (ZnO3), Supplementary Fig. S8. Every pixel of the POLYPHEME maps performed has a significant contribution of the 380 nm emission, even for those with higher intensity emissions above 400 nm, confirming the ubiquitous presence of this pigment in the samples. The ratios between the three bands varied from pixel to pixel, in the same sample and within samples, showing the heterogeneity of this ZnO-polymer systems, Fig. 7 and Supplementary Figs. S8–S15. However, for each of the objects studied, characteristic trends were observed, which will be discussed below.

The ZnO band at ca. 380 nm does not overlap with the CN emission, Fig. 5C. On the other hand, considering that these objects have an estimated camphor concentration between 20 and 15% w/w, Supplementary Fig. S12, the emission of this plasticizer at ca. 420 nm can contribute to an intensity increase in this region.

Overall, the four objects analyzed showed different emission profiles: in the 1901 postcard the emission at 510 nm (ZnO3) dominates over the other two bands; the 1899 calendar is characterized by a highly heterogeneous system with high-intensity emissions from the three bands; for the Holy Bible Pin and the American Flag Pin, the band at 380 nm displays the highest intensity, Fig. 7 and Supplementary Figs. S13–S15.

The American flag advertisement pin mappings were characterized by the strong emission of zinc oxide particles, Fig. 7. The heterogeneity of the system was observed on images collected with the full field endstation TELEMOS, by designating colors to each emission bandpass filter used: blue for the 352–388 nm range to image zinc oxide (ZnO); green for the 412–438 nm range to image ZnO trapped states, cellulose nitrate degradation, camphor, or other admixed materials; and red for the 535–607 nm range to image the ZnO trapped states and cellulose nitrate degradation, Fig. 7. In future work, we will investigate the origin of these differences by studying a set of aged and unaged reference samples. In this work, we will examine in more detail the American Flag Pin to better understand the information it is possible to extract based on the luminescent properties of ZnO, CN and camphor. The results correlated with POLYPHEME mapping in which we observed, spatially, spectral variations related to the main emission features, Fig. 7C. These emission features can be represented by three average spectra (calculated from 10 selected pixels) and are characterized by (1) 380 nm emission, accounting for 67% of the mapped area, (2) emissions between 400 and 450 nm (10%) and (3) ZnO trapped state emission above 500 nm (23%), Fig. 7D, Supplementary Figs. S9 and S10. To better understand the causes of these variations, preliminary Raman and infrared analyzes were carried out. The main results are summarized in Supplementary Table S1. μRaman analysis of whitish particles in the American flag pin, studied by TELEMOS and depicted with a strong blue luminescence in Fig. 7, confirmed the presence of ZnO by its characteristic peaks at 330 and 475 cm^−1^ and identified a peak at 1054 cm^−1^ indicative of cerussite (PbCO_3_), Supplementary Fig. S11 and Table S1. This also agrees with the identification of Zn, Pb and Ca by EDXRF. Zinc stearate was also detected by Raman peaks at 1062, 1094, 1127, 1296 and 1445 cm^−1^, its presence is confirmed by FTIR-ATR by comparison with a reference, Supplementary Figs. S11 and S12. It was also possible to identify peaks related to the polymer matrix, namely the nitrate groups (864, 1286, 1652 cm^−1^) and camphor (650 cm^−1^) main vibration. Zinc stearate and cerussite are compounds that can be responsible for the variations observed between the ZnO1 emission at 385 nm and the ZnO3 trapped state broad emission between 500 and 550 nm^40,46,49,50^, Fig. 7. Emissions between 400 and 450 can be due the ZnO defect-associated emissions, camphor, or localized cellulose nitrate degradation^46,48^. Future work will investigate more in-depth the origin of these emissions and if they can be related to specific formulations of ZnO that could be used as a signature to a technological process (for colour production).

In summary, it was possible to visualize at the macro and sub microscale, the heterogeneity of these complex mixture of ZnO with celluloid. Three spectral regions of interest were identified: the ZnO1 (385 nm) and ZnO3 (500–550 nm) emissions whose intensity variations can be influenced by the presence of organic functionalized ZnO and other inorganic additives or impurities; and the 400–450 nm regions whose contributions can be due to ZnO crystal defects (ZnO2) or to the polymer associated emissions, either camphor or cellulose nitrate degradation. Comparing POLYPHEME results for the four objects analyzed it was possible to observe differences in their spectral signatures and spatial distribution at the submicrometric scale, Fig. 7 and Supplementary Figs. S13–S15. The different emission features of these two objects can be due to formulation differences, shown by Raman analysis, which identified massicot/litharge (PbO) and zinc stearate for the 1901 postcard and anatase (TiO_2_) and azurite for the holy bible pin, Supplementary Table S1. These results will be further explored in future work, but already show the potentiality of this technique to distinguish color formulations that can also be linked with the three different ZnO manufacturing methods employed in the late nineteenth early twentieth centuries^40,46,51^.

## Conclusions

The present study here describes the implementation of synchrotron deep UV photoluminescence micro spectral-imaging for investigating (DUV-µPL), at the sub-microscale, cellulose nitrate-based objects' material heterogeneity that correlates to its history (of alteration and manufacture). The investigation of cellulose nitrate and celluloid luminescence properties using DUV excitation has revealed the presence of markers that can be used for the detection of the degradation process at the early stage. By using excitation between 250 and 300 nm, DUV-µPL has allowed visualizing the distribution of degradation products in cinematographic films, with very high resolution, providing new insights into their degradation mechanisms. During degradation, the emission spectra of cellulose nitrate in the UV–VIS modifies markedly; the initial low emission at 420 nm increases and a new band at 510–520 nm appears. The higher the intensity of the band at 510–520 nm over the first band (*ca.* 420 nm), the higher the extent of degradation. Importantly, we proved that the evolution of the ratio between the two bands (I_425nm_/I_510nm_) correlates with DS calculated by infrared spectroscopy, increase in this ratio with decrease in DS, showing that it is possible to monitor the level of degradation of CN by DUV-µPL. This tool allowed another important discovery: the higher the camphor concentration the higher the degradation rate both in reference samples and on cinematographic films. This crucial information will be next tested within an extended set of historical objects possibly using a chemometrics approach. Future experiments will also test an extended set of CN and celluloid samples, aged with shorter irradiation times, to better discuss the chromophores that give rise to the emission bands at 425 and 510 nm. At this point, it is not possible to attribute them to the excited sate emission of the key intermediates formed during degradation: alcohols, hydroperoxides or lactones depicted in Fig. 3.

We also demonstrate the asset of these techniques to characterize and map, by combining spectral and spatial information, the heterogeneous microenvironments of the zinc oxide-based pigment in celluloid heritage. Due to the intrinsically heterogeneous nature of the objects in the Perlov collection, the variations observed in the luminescence of zinc oxide could be linked to the presence of lead carbonate or white lead (identified by Raman microscopy), and as such to a specific color formulation^46^. Overall, this study shows that DUV-µPL, due to its unique detection limit and high spatial resolution, is a fundamental technique for the study of cellulose nitrate-based heritage and plastic heritage, in general.

Finally, this work shows how the collection of celluloid objects donated by Perlov is a valuable historical source. Each object offers a biography that represents a valuable way of informing its past. The object's biography is a necessary research tool to understand and contextualize the relationships between people and the meaning, purpose, and use of an object. It is also an effective didactic tool used in museums for teaching history. To write these biographies, POLYPHEME and TELEMOS have the potential to provide important data on manufacturing particularities. Knowing more about the object’s materiality is also necessary to better preserve it in a historical and material sense.

## Methods

### Cinematographic films and objects from Perlov's collection and sample preparation

The cinematographic films were provided by the Austrian Academy of Sciences and the German Film Institute, partners of the NEMOSINE project. The selected celluloid objects belong to the Perlov’s collection, a collection that consists of 300 everyday celluloid objects, donated by Amy Schenkein and Dadie Perlov, American collectors and majority donors of the Smithsonian Institute's celluloid collection.

Cross-sections of the cinematographic films were prepared by using a Leica RM 2155 rotary microtome equipped with low profile blades Leica DB 80 LX. To do so, a small sample was removed from the border of the cinematographic films (about 2 × 2 mm). This fragment was taped over a small piece of polymethylmethacrylate (PMMA) to hold it steady (the glue did not enter in contact with the sampling area). The PMMA was previously cleaned with ethanol. This assembly was fixed in the microtome clamping system. 15 μm slices were cut by using a clean portion of the blade for each cut and making a quick but relatively gentle motion (to get a clean cut and avoid ridges and fractures). The cuts were controlled by using a stereomicroscope. The sample cross-sections were placed over a microscope slide. This method allows the proper observation of the stratigraphic layers of the cinematographic films without the use of embedding resins.

Cross-sections of the celluloid objects were prepared by microsampling using Ted Pella μ-tools and a Leica MZ16 stereomicroscope. Since the objects studied have thicknesses below 1 mm, the micro samples were cut from the corners of the objects from one end to the other (cross-section). The cross-sections were embedded in a polyester resin (Clear Casting Polyester Resin AM) so that the areas in contact with the resin corresponded to the surface of the object and the interior of the cross-section to the object’s bulk. The samples were wet ground on a polishing wheel to expose the cross-section. Micro-Mesh® sheets with grit 8000 were used for dry polishing.

### Reference samples preparation

Cellulose nitrate films were obtained from a solution of 4% (w/v) in methanol, prepared at room temperature (20 °C) and allowing cellulose nitrate to dissolve through the night (approx. 12 h). This solution was homogeneously poured over a porcelain vessel using a Pasteur pipette and placed inside a desiccator with silica gel. The solution was left drying for 3 h. After drying, a transparent cellulose nitrate film was peeled off the vessel with Ted-Pella micro tweezers. Samples with an area of 2.5 × 1 cm^2^ were cut with a scalpel. The thickness of these reference films was 150 µm (approaching the thickness of the polymeric support of a cinematographic film). Celluloid films were obtained by adding camphor to the previous solution, in a ratio of 70/30 (cellulose nitrate/camphor) in weight. They were prepared following the same methodology used for the cellulose nitrate films.

### Artificial ageing

The irradiation of the cellulose nitrate and celluloid reference films and the cinematographic film DIF 50 500 (cut in pieces with dimensions of ca. 2 × 1 cm^2^) was carried out in a CO.FO.ME.GRA accelerated aging apparatus (SolarBox 3000e) equipped with a Xenon‐arc light source, an outdoor filter λ ≥ 280 nm, with constant irradiation of 800 W/m^2^ and a temperature of 40 °C. The films were irradiated for a maximum period of 150 h (total irradiance = 365 MJ/m^2^). To avoid sample movement inside the irradiation apparatus, the samples were placed inside “homemade” glass boxes with a quartz plate over (100 mm × 100 mm × 3 mm thick, UQG Optics Limited).

### Instrumentation

#### Optical microscopy

Microsampling of the celluloid objects was performed with Ted Pella μ-tools using a Leica MZ16 stereomicroscope. This microscope has a 7.1 × to 115 × zoom range lens, equipped with an integrated Leica ICD digital camera and a Leica KL 1500 LCD external cold light source with two flexible optic fibre cables. Microphotographs of the cinematographic cross-section were acquired using a Axioplan 2ie Zeiss microscope equipped with 10 × ocular lenses and a 20 × Epiplan objective, an incident halogen light illuminator (tungsten light source, HAL 100) and a digital Nikon camera DXM1200F, with Nikon ACT-1 software.

#### Spectrofluorimetry

Cellulose nitrate excitation spectra were acquired using a Jobin Yvon/Horiba SPEX Fluorog 3-2.2 spectrofluorometer with a 450 W xenon lamp and a double-grating monochromator. Measurements with the spectrofluorometer were performed at front-face (ff). In this technique, the incident excitation beam is focused on the front surface of the samples and the fluorescence emission is acquired from the same region at an angle of 22.5°, which minimizes reflected and scattered light. To ensure that different samples were analysed in the same area, the reference films were placed on quartz demountable cells (Lightpath Optical (UK) Ltd.) and mounted in a cell holder for short path length cells (Lightpath Optical (UK) Ltd.), with the film surface directly facing the beam. Corrected emission and excitation spectra were collected using 3 mm entrance and exit slits, an integration time of 0.2 s, an increment of 2 nm. Excitation spectra were recorded collecting the signal at 390, 450 and 480 nm. To ensure the reproducibility of the results, 2 sets of four samples of irradiated cellulose nitrate and celluloid were analyzed.

#### UV–VIS spectroscopy

Ultraviolet–visible absorption spectra were recorded on a Varian-Cary 100 Bio spectrophotometer, between 200 and 800 nm with air as a reference and a constant temperature of 20 ± 1 °C. To ensure that different samples were analysed in the same area, the reference films were placed on quartz demountable cells (Lightpath Optical (UK) Ltd.) and mounted in a cell holder for short path length cells (Lightpath Optical (UK) Ltd. To obtain absorbances < 2, the films were prepared with thickness < 10 μm using solutions of 1.85% (w/v) in methanol applying the methodology described in “Reference samples preparation” section.

#### µInfrared spectroscopy

All samples were analyzed by µFourier Transform Infrared Spectroscopy for molecular characterization of the polymer matrix. Infrared spectra were acquired on a Nicolet Nexus spectrophotometer equipped with a Nicolet Continuμm (15 × objective) microscope and a Mercury–Cadmium–Tellurium (MCT) detector cooled by liquid nitrogen. Micro samples were placed on a diamond cell and the spectra were acquired in transmission mode between the 4000–650 cm^−1^, with a resolution of 8 cm^−1^ and 128 scans. Spectra are shown as acquired, without corrections or any further manipulation, except for the removal of the CO_2_ absorption at approximately 2300–2400 cm^−1^.

#### µRaman microscopy

Celluloid cross-sections were analyzed by µRaman to obtain further information on inorganic additives in correlation with Micro‐energy dispersive X‐ray fluorescence analysis. The Raman spectra were collected on a Labram 300 Jobin Yvon spectrometer equipped with a He‐Ne laser operating at 632.8 nm. The laser beam was focused with an Olympus 100 × lens with a spot size of 2 μm. The laser was used at maximum power (without the use of filters), with a collection time of 15–20 s performing 20 to 30 scans. It was used a high-resolution grating (1800 grooves/mm).

#### Micro‐energy dispersive X‐ray fluorescence

X‐ray fluorescence of the celluloid objects was performed in situ with a Bruker ArtTAX Pro spectrometer equipped with a Molybdenum (Mo) ampule, Peltier effect cooled Xflash 3001® semiconductor detector and a movable arm. The experimental parameters used were voltage of 40 kV, current of 300 μA, acquisition time of 180 s.

#### Micrometer

Sample thickness was measured using a TOPEX 31C629 micrometer, which has a length measurement from 0 to 25 mm and an accuracy of 10 ± 5 µm.

### Synchrotron deep UV-excited photoluminescence

Samples were analyzed at the POLYPHEME and TELEMOS end-stations of the DISCO beamline (Synchrotron SOLEIL, Gif-sur-Yvette, France). POLYPHEME was used to perform hyperspectral photoluminescence micro-imaging and acquire full emission spectra with high spectral resolution; TELEMOS for full-field luminescence microscopy^39,52^.

#### POLYPHEME

Emission spectra were recorded on an Olympus IX71 inverted microscope with lens replacement to be transparent in the deep UV range, a 40 × Zeiss Ultrafluar UV-transmitting immersion objective, with monochromatic excitation wavelengths of 290 and 330 nm, an emission wavelength range of 320–640, using integration times between 5 and 20 s per spectrum. We tested our experimental conditions and found that no degradation is induced in the samples, with the excitation beam; thus, we did not observe changes in the fluorescence of the references after repeated irradiation at 290 nm and 330 nm in the same point of analysis. Raster scanning maps were performed with integration times of 5 s (2 accumulations) and 10 s (1 accumulation), in areas between 280 µm^2^ and 1.25 mm^2^ using 2 to 15 µm steps. In Fig. 5C, the emission data was normalized to the range 0 to 1 applying the function *normalize to* [0, 1] $\left( {y = \frac{{y - y_{\min } }}{{y_{\max } - y_{\min } }}} \right)$ using Origin2016 Pro software.

References and cinematographic cross-sections were analysed between two 170 μm quartz coverslips. Embedded celluloid cross-sections were placed on top of a 170 μm quartz coverslip. During the experiments, the in-situ analysis of the 1899 calendar was tested with success.

Data acquired were pretreated in Labspec for the removal of cosmic spikes using a top-hat filter and map colour assignments. For the quantification of the emission contributions for each pixel in the American flag pin POLYPHEME mapping, Fig. 7C, a direct classical least squares (DCLS) modeling using Labspec 5 was performed. Three average spectra, each one calculated from 10 spectra characterized by (1) strong near band edge emission (blue loading), (2) ZnO crystal defect emissions between 400 and 450 nm (green loading), and (3) strong green band emission (red loading), were used as reference component spectra (loadings) Fig. 7D. For each pixel, the model finds a linear combination of the reference component spectra which best fits the raw data. Using three loadings (blue, green, and red) with scores (x, y and z) the sum, S, of the linear combination is represented by: S = [x * blue] + [y * green] + [z * red]. The model provides the scores in percentage and data was normalized so that the combination of all scores adds to 100%. The bigger the score the bigger the similarity with the loading. An example of the model output for an emission spectrum is given in Supplementary Fig. S9. This procedure was used to identify the distribution of the reference component spectra within the spectral array to create a profile based on each component distribution, Supplementary Fig. S10. Based on the scores obtained for each pixel (448 pixels out of a total of 560 due to resin emission, not showed in Fig. S2) the total averages were calculated for the entire map. Blue loading accounted for 67% of the mapped area, the green loading accounted for 10%, *a*nd the red loading for 23%. The error map for this model is showed in Supplementary Fig. S16.

#### TELEMOS

Images acquired were collected using an Axio ObserverZ1 microscope (Carl Zeiss MicroImaging) with a 40 × Zeiss Ultrafluar UV-transmitting immersion objective, with a monochromatic excitation wavelength of 290 nm and a dichroic mirror with a cut-off wavelength at 300 nm. Fluorescence was collected using four emission bandpass filters: 352–388 nm; 412–438 nm; 535–607 nm. Acquisition time was set at 10 s for all channels. Images obtained were treated with ImageJ software: a BASIC corrected DW processing was used to remove the artefacts; the false-color images were created by associating a designated color to each channel (blue, green, and red) and manipulating the color balance. This method allowed localizing areas with the highest fluorescent signals.

## Supplementary Information

Supplementary Information.

## Data Availability

All data needed to evaluate the conclusions in the paper are present in the paper and/or the Supplementary Materials. Additional data related to this paper may be requested from the authors.
