# Supplementary material for: Novel﻿ markers to early detect degradation on cellulose nitrate-based heritage at the submicrometer level using synchrotron UV–VIS multispectral luminescence
Source: Sci Rep. 2021 Oct 12;11:20208. doi: 10.1038/s41598-021-99058-6 (PMC8511177; doi:10.1038/s41598-021-99058-6)
Supplement: Supplementary file 1 — Supplementary Information. [file 41598_2021_99058_MOESM1_ESM.pdf]

Supplementary Information

Novel markers to early detect degradation on cellulose nitrate-based heritage at the submicrometer level using synchrotron UV-Visible multispectral luminescence

Artur Neves[1], Ana Ramos[1], Maria Elvira Callapez[2], Robert Friedel[3], Matthieu Réfrégiers[4,5], Mathieu Thoury[6,*] & Maria João Melo[1,*]

[1]LAQV/REQUIMTE, Department of Conservation and Restoration & of Chemistry, NOVA School of Science and Technology, Universidade NOVA de Lisboa, 2829-516 Caparica, Portugal

[2]Centro Interuniversitário de História das Ciências e da Tecnologia, Faculdade de Ciências, Universidade de Lisboa, Campo Grande, Lisbon, 1749-016, Portugal

[3] Department of History, University of Maryland, College Park, MD 20742, USA

[4]Synchrotron Soleil, l'Orme des Merisiers, BP48 St. Aubin, 91192 Gif-sur-Yvette, France

[5]Centre de Biophysique Moléculaire, CNRS UPR4301, Rue Charles Sadron, 45071 Orléans, France

[6]IPANEMA, CNRS, Ministère de la Culture, Université de Versailles Saint-Quentin-en-Yvelines, Université Paris-Saclay, BP48 St. Aubin, 91192 Gif-sur-Yvette, France

*Author to whom correspondence should be addressed: mjm@fct.unl.pt

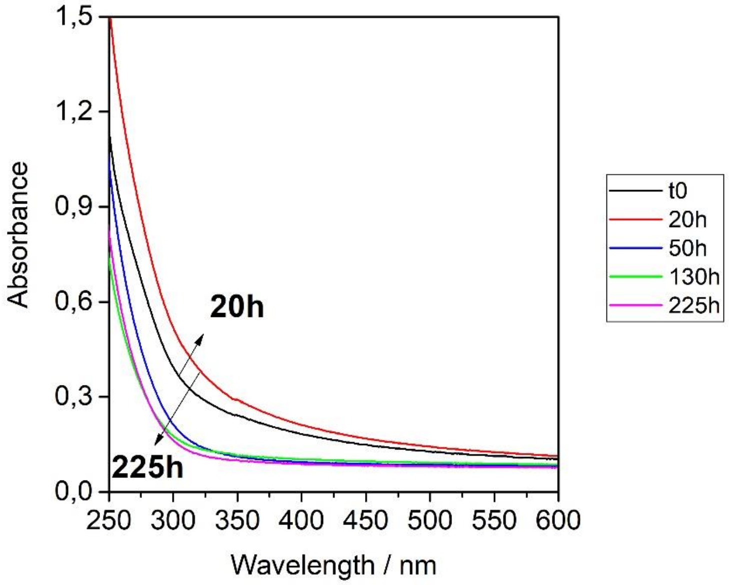

Figure S1. UV-VIS absorption spectra of cellulose nitrate films (ca. 1µm), irradiated at 20h, 50h, 130 and 225 (λ_{irr} ≥ 280nm, 60ºC).

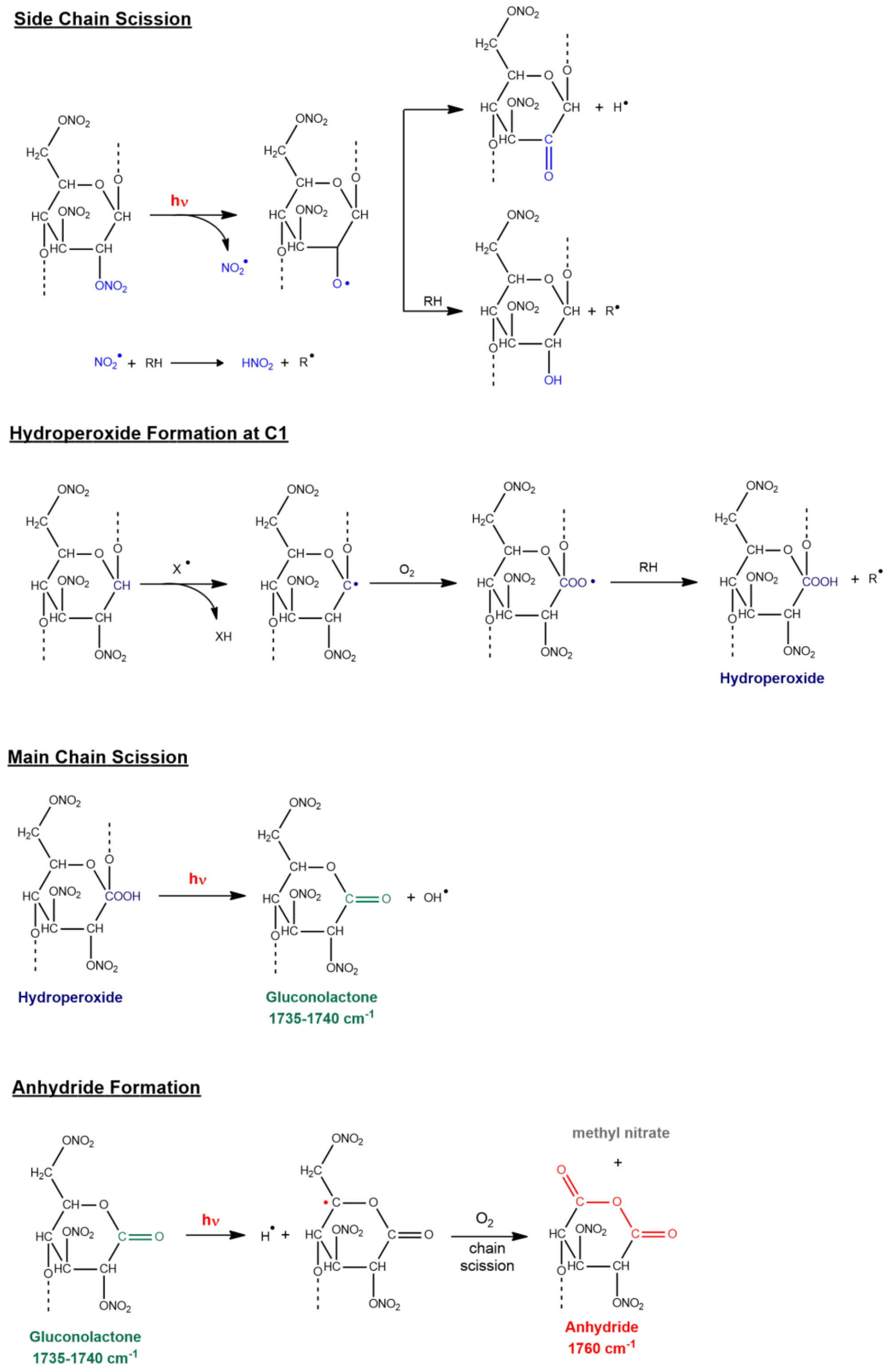

Figure S2. Cellulose nitrate side-chain scission starts with de-nitration, by the homolysis of a nitrate group in C2 or C3 and the release of $^{\bullet}NO_2$. The alkoxy radical formed can be converted into a ketone or hydroxyl group. $X^{\bullet}$ is defined as any radical which will promote $H^{\bullet}$, namely $^{\bullet}NO_2$ or $RO^{\bullet}$, which lead to the formation of nitrous acid (HNO_2) or a hydroxyl group (ROH) respectively. C1H is the most labile hydrogen and thus the most easily abstracted. **Hydroperoxide formation at C1** will occur, in the excited state, by reaction of the produced macroradical with oxygen and further $H^{\bullet}$ abstraction. This leads to the formation of the hydroperoxide and to the formation of another macroradical $R^{\bullet}$ in the cellulose nitrate structure. ***Cellulose nitrate main chain scission*** occurs due to the decomposition of the excited state hydroperoxide, leading to β scission of the glycosidic bond and formation of a gluconolactone (detected in the infrared at $1735cm^{-1}$-$1740cm^{-1}$). ***Anhydride formation*** will occur in a second step, following light absorption, in which the macroradical produced at C5 will react with oxygen, leading to the formation of a carbonyl at C5 and the release of $^{\bullet}CH_2ONO_2$, which converts into a final product, methyl nitrate, by $H^{\bullet}$ abstraction. This anhydride function if detected in the infrared at $1760cm^{-1}$ [19,26]. For more details on the degradation mechanism of CN, please see p. 144 in ref. 26.

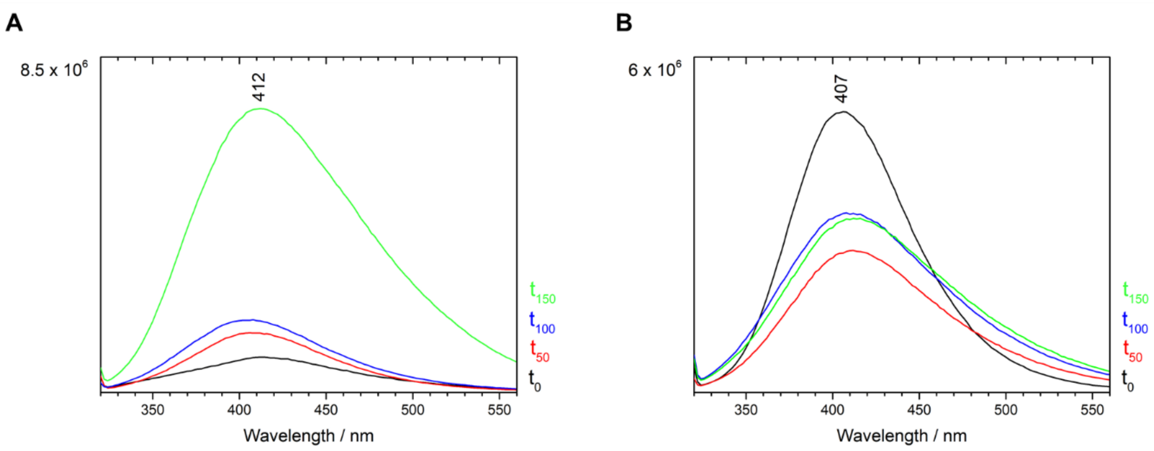

Figure S3. Emission spectra (λ_{exc} = 290nm) of artificially aged references irradiated during 50h, 100h and 150h ($\lambda_{irr} \geq$ 280nm, 60ºC), using a spectrofluorometer, in **A)** cellulose nitrate and **B)** celluloid (70% cellulose nitrate and 30% camphor w/w).

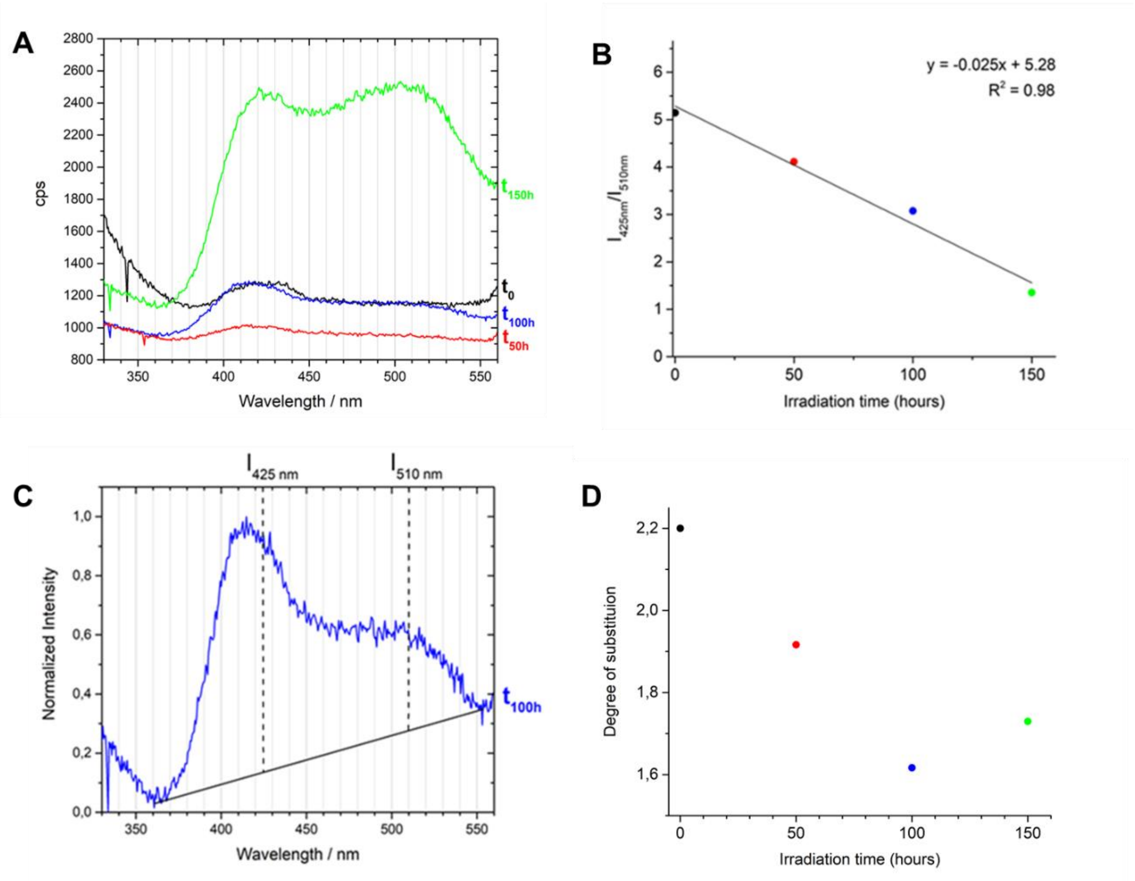

Figure S4. A) Emission spectra (λ_{exc} = 290nm) of artificially aged cellulose nitrate irradiated during 50h, 100h and 150h. **B)** The ratio I_{425nm}/I_{510nm} was plotted over irradiation time and a linear regression was calculated. **C)** I_{425nm} and I_{510nm} were calculated as exemplified for t_{100h}, the intensities were corrected for each spectrum by applying a baseline between 360 nm and 550 nm. **D)** The degree of substitution of the artificially aged cellulose nitrate references was plotted over the irradiation time. At 150h of irradiation, main chain scission leads to the decrease of the νCOC band (1070 cm^{-1}) which is the reference band used for the DS calculation, resulting in higher DS values. Although this measuring technique shows this limitation for high degradation times, bellow 100h of irradiation the expected DS decrease was observed. In the future, shorter irradiation time intervals will provide more details on how DS correlates with the emission of cellulose nitrate at early stages of degradation (< 100h).

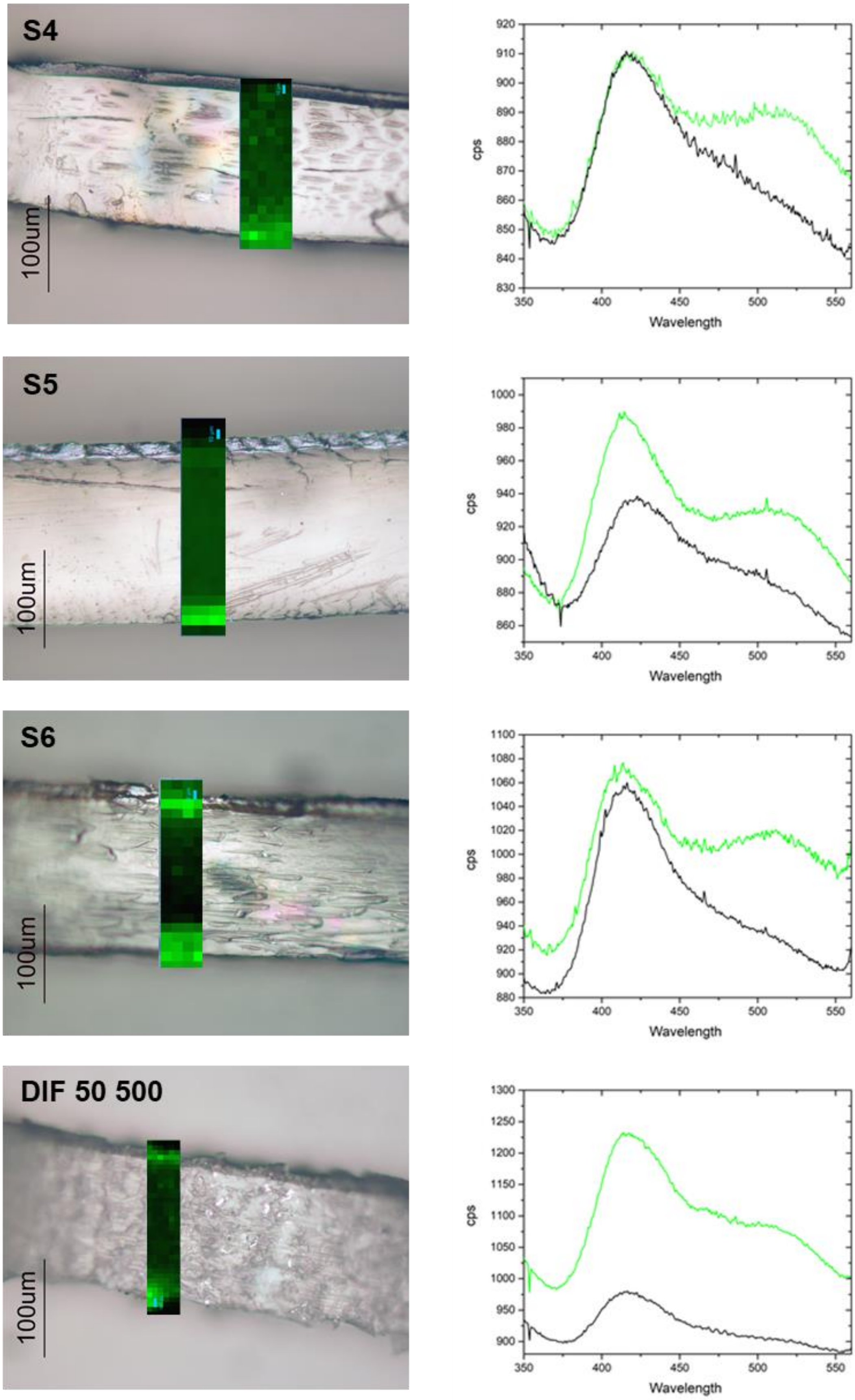

Figure S5. POLYPHEME raster scan mapping of cinematographic films S4, S5, S6 and 50509. The maps show the intensity variation of the emission band between 500 and 550 nm. Emission spectra collected at the interfaces (green) and the interior (black) of the cross-sections are shown.

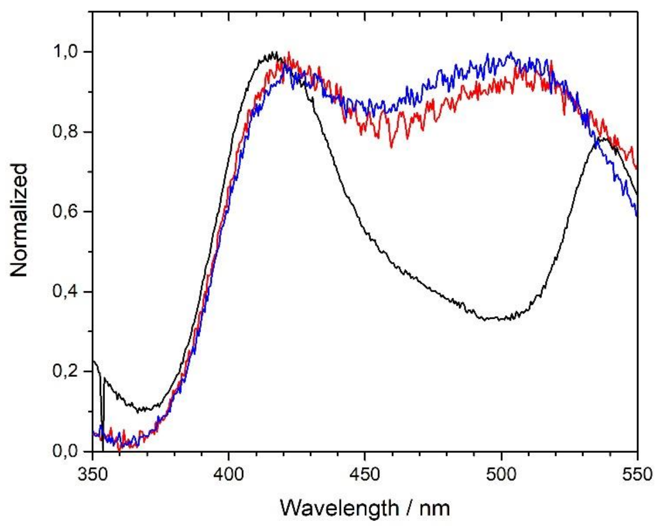

Figure S6. Normalized emission spectra of DIF 50 500 cellulose nitrate support at t_0 (black line) and 150h of irradiation (red line). The spectra are compared with the emission spectrum of cellulose nitrate artificial aged reference irradiated during 150h (blue line).

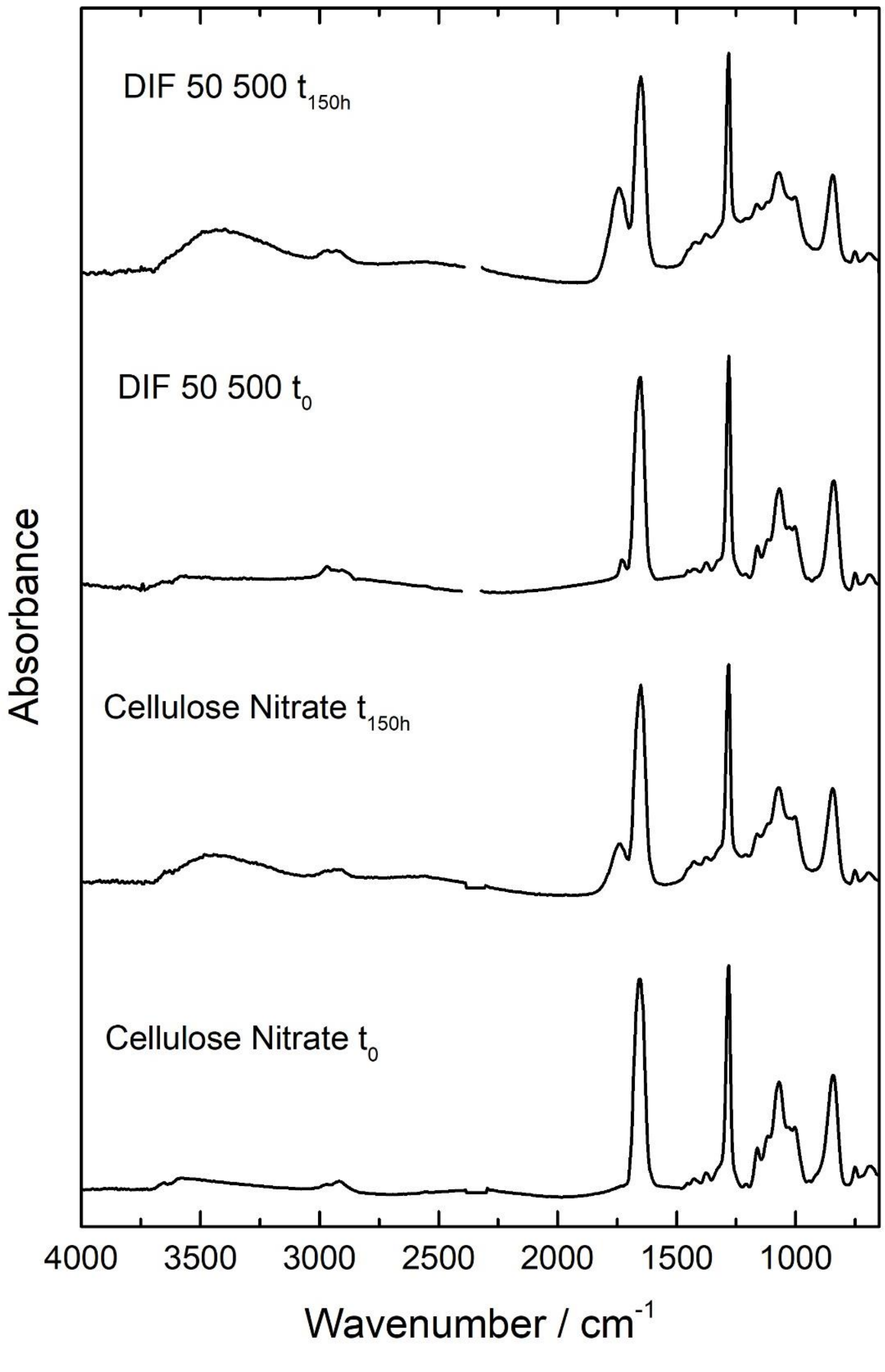

Figure S7. Infrared spectra of unaged and aged (150 hours, λ_{irr} ≥ 280nm, 40°C) cellulose nitrate references and cinematographic film DIF 50 500 samples. Using Bussiere et al (2014) calibration curve for the quantification of camphor (A_{1730}/A_{1655} = 0.013 x %camphor, in a rough approximation without considering the degree of substitution), in DIF 50 500 we found 6% camphor w/w.

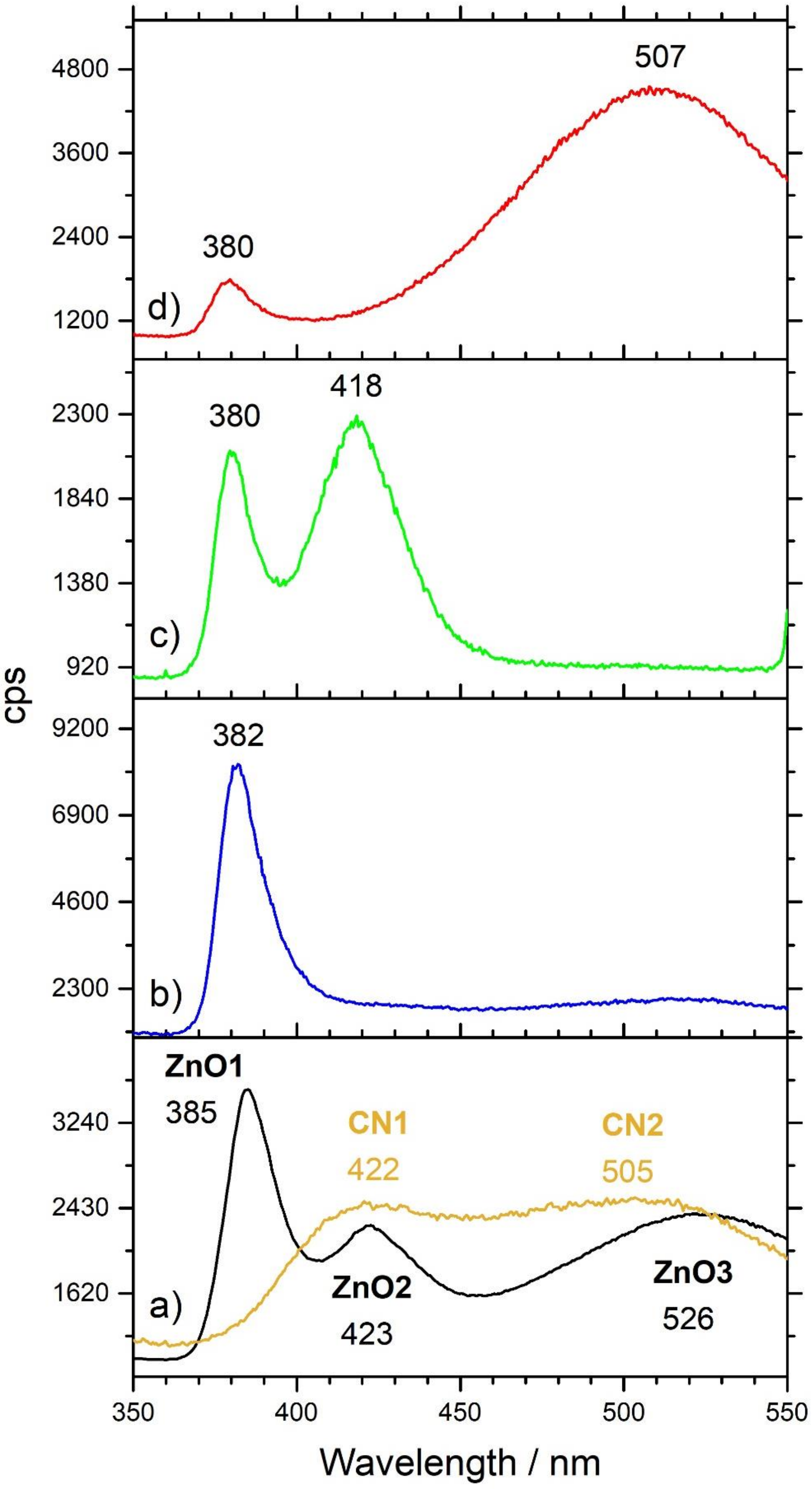

Figure S8. a) Emission spectra of zinc oxide from the American Flag Pin (black line) and of a 150h artificial aged cellulose nitrate reference sample (orange line). The ZnO1 band is the band edge emission of ZnO particles (380-385nm); the ZnO2 band/region is due to the ZnO crystal defect emissions between 400 and 450 nm; the ZnO3 broad band is the green emission from ZnO particles; the CN1 band (422nm) is the band that characterizes cellulose nitrate; the CN2 broad band (with max between 500-520 nm) is the band which intensity correlates with cellulose nitrate degradation. **b)** Emission spectra of a ZnO1 band with very high intensity obtained from the holy bible pin microsample. **c)** Emission spectra of a high intensity ZnO2 band in comparison to ZnO1, obtained from the 1899 calendar microsample analysis. **d)** Emission spectra of a ZnO3 band with very high relative intensity in comparison with ZnO1, obtained from the 1901 postcard analysis.

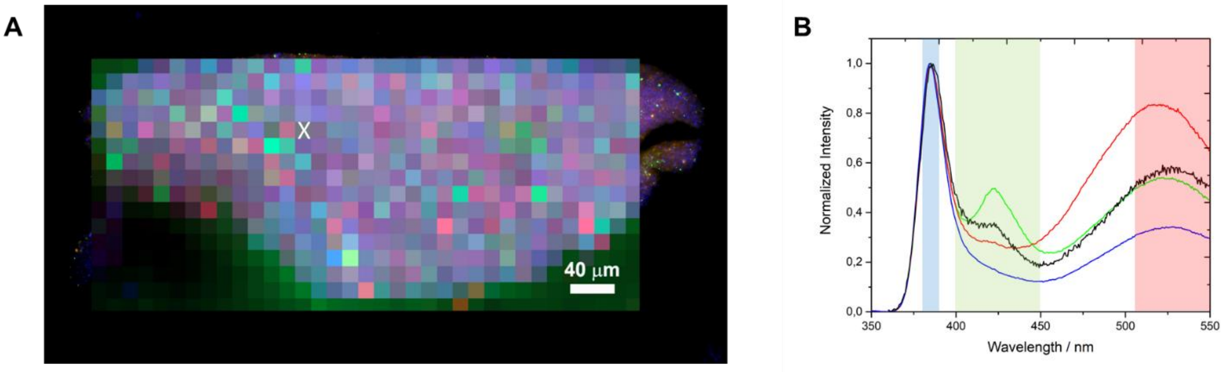

Fig. S9. A) Raster scanning map by POLYPHEME (15 x 15 μm^2, 5s, 2 accumulations, λ_{exc} = 290 nm) of the American flag celluloid advertisement pin. Three spectral regions were used to map the emission spatial distribution, shown in **B)**. Colors were designated as follows: blue for the band edge emission at 385 nm, green for the spectral region between 400-450 nm; red for the region between 510-550 nm. **B)** Average spectra, calculated from the selection of 10 spectra from A), were used as reference component spectra (loadings) to quantify the emission contributions of these spectral regions for each pixel in a direct classical least squares (DCLS) model. For more details, please see text. As an example of the output given by the model for one pixel, the emission spectrum of the marked pixel (X) in map A) is shown (black spectrum). For this spectrum, the model gave a match of 42.8% for the green loading, 28.9% for the blue loading and 28,5% for the red loading.

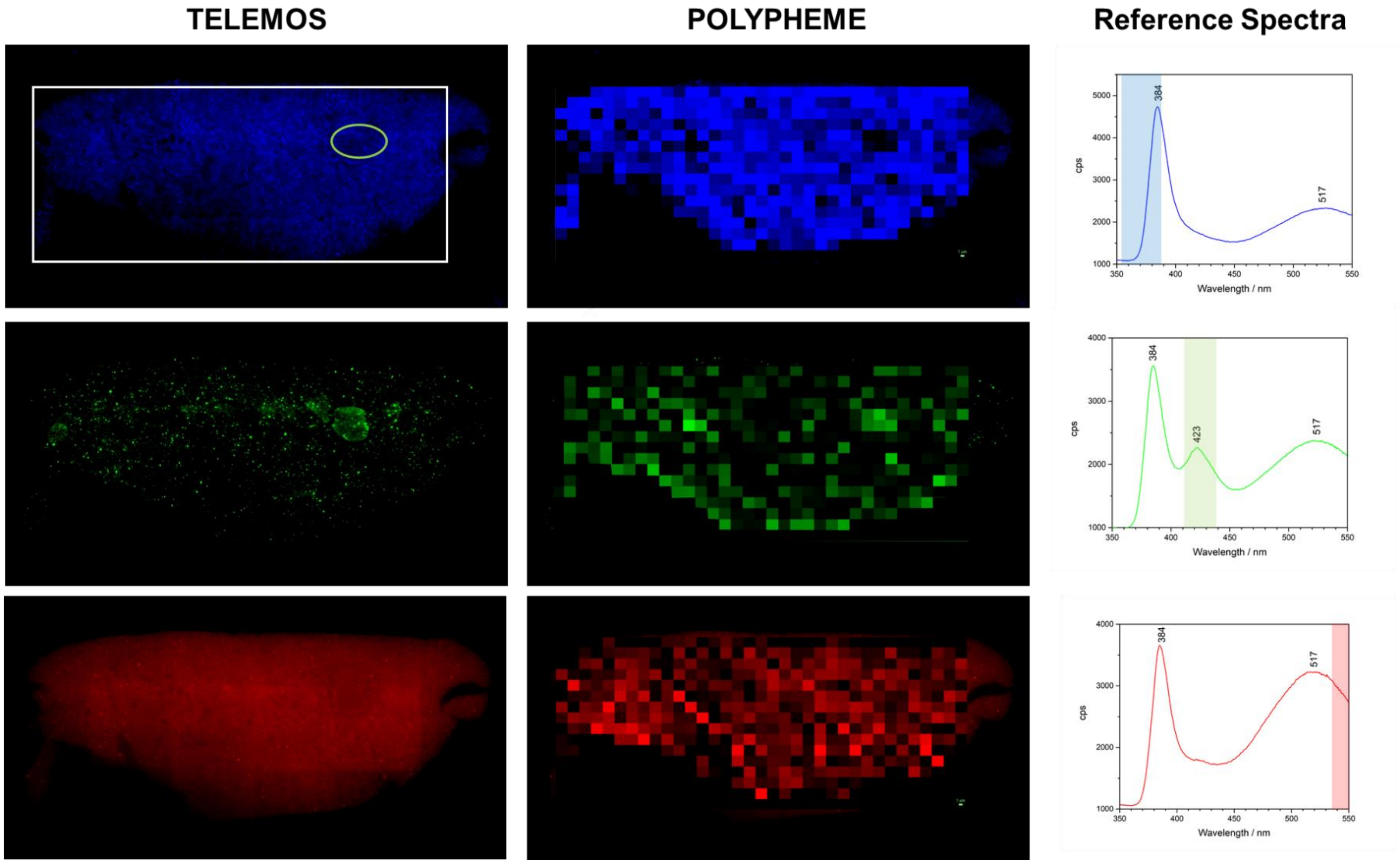

Figura S10. TELEMOS, full-field luminescence imaging of the American flag celluloid advertisement pin (λ_{exc} = 290 nm, 40x objective). Emission bandpass filters used: 352-388 nm (blue); 412-438 nm (green); 535-607 nm (red). The white-square marks indicate the POLYPHEME map area; **POLYPHEME**, emission distribution profiles for each reference component spectra used in the direct classical least squares (DCLS) modeling of the spectral array. For more details, please see text. It is interesting to observe that the area marked by the green ellipse on the TELEMOS mapping correlates with the POLYPHEME which shows a higher score of the green loading in comparison to blue loading; **Reference component spectra** used in the model, each one obtained for the average of 10 selected POLYPHEME emission spectra. The brighter the pixel on the distribution profile, the higher the correlation with the reference component spectra. The spectral regions viewed using TELEMOS full-field luminescence imaging setup are highlighted, with colors corresponding to the bandpass filters used.

Table S1. µRaman main results for the celluloid object micro samples analysed. The degree of substitution (DS) was calculated with µFTIR using the calibration curve described in Nunes et al. (2020).

Object	Date	FTIR DS	Type of sample	µRaman main results
Postcard 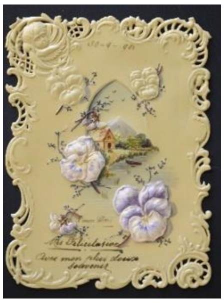	Possibly from 1901	2.06	micro-sample embedded in polyester resin	**Cellulose nitrate**: 856, 1286, 1650, 2932 and 2970 cm^{-1}<br>**Camphor**: 654 and 1730 cm^{-1}<br>**Zinc stearate**: 1065, 1131, 1297, 1441,1458, 2851 and 2884 cm^{-1}.<br>**Massicot and litharge** (PbO): 89, 108, 125 and 141 cm^{-1}
American flag Pin 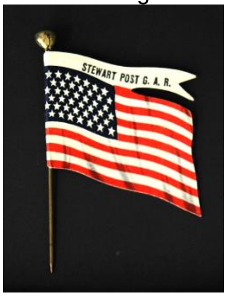	N.A.	2.01	micro-sample embedded in polyester resin	**Cellulose nitrate**: 864, 1286, 1652, 2932 and 2970 cm^{-1}<br>**Camphor**: 650 and 1730 cm^{-1}<br>**Zinc Oxide (ZnO)**: 330 and 475 cm^{-1}<br>**Cerussite ($PbCO_3$)**: 1054 cm^{-1}<br>**Zinc stearate**: 1062, 1094, 1127, 1296 and 1445 cm^{-1}
Holy Bible Pin 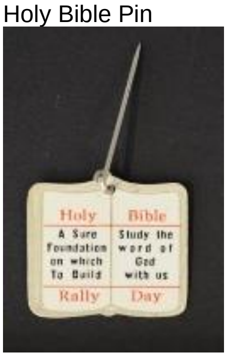	N.A.	2.00	Micro-sample embedded in polyester resin	**Anatase** (TiO_2): 257, 547, 583, 808, 1093 cm^{-1}<br>**Azurite**: 141, 394, 512, 637 cm^{-1}
Calendar 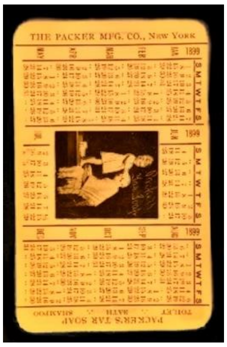	1899	1.88	Micro-sample embedded in polyester resin	**Cellulose nitrate**: 862, 1284, 1653 cm^{-1}<br>**Camphor**: 650 and 1733 cm^{-1}<br>**Zinc Oxide (ZnO)**: 475 cm^{-1}<br>**Lead chromate ($PbCrO_4$)**: 145, 321, 341, 379, 824, 837 and 846 cm^{-1}

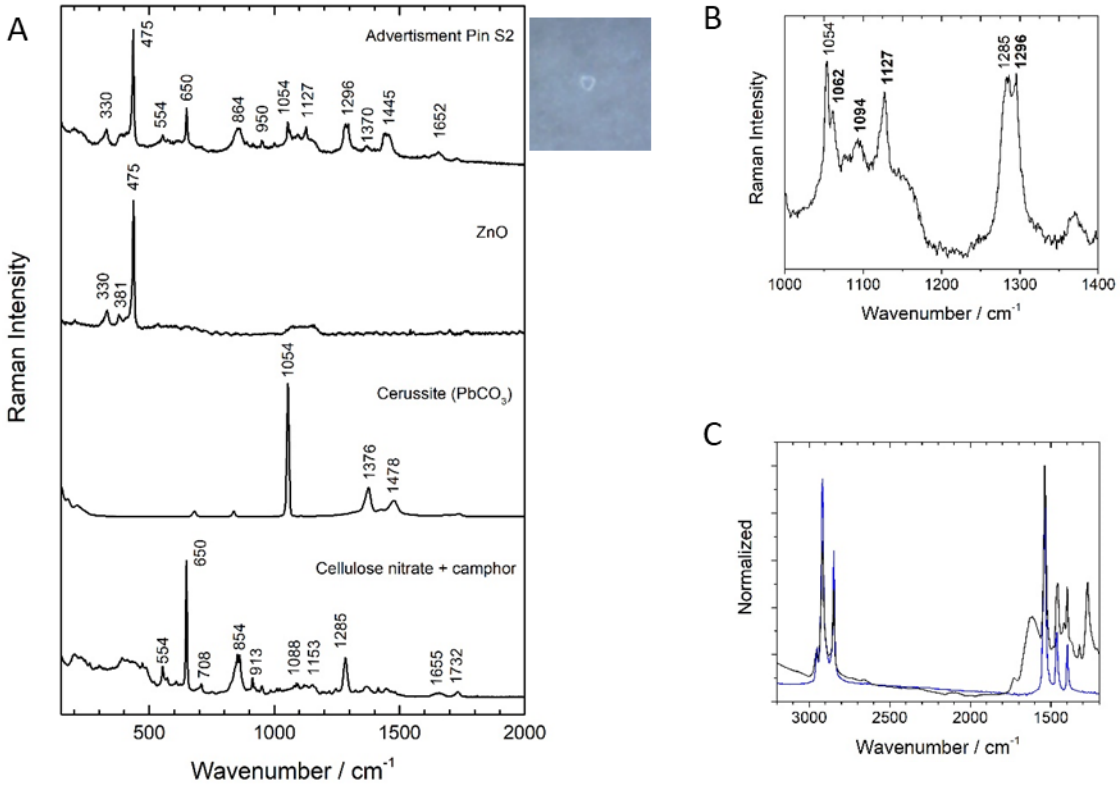

Figure S11. A) Raman spectra of a whitish particle in the American flag pin sample (633 nm laser) and references of zinc oxide, cerussite ($PbCO_3$) and celluloid (cellulose nitrate and camphor). **B)** Raman spectrum in the spectral region between 1000 and 1400 cm^{-1}. Peaks attributed to zin*c* stearate are emphasized in bold. **C)** FTIR-ATR spectra of the American flag pin (black) and of a zin stearate reference (blue)

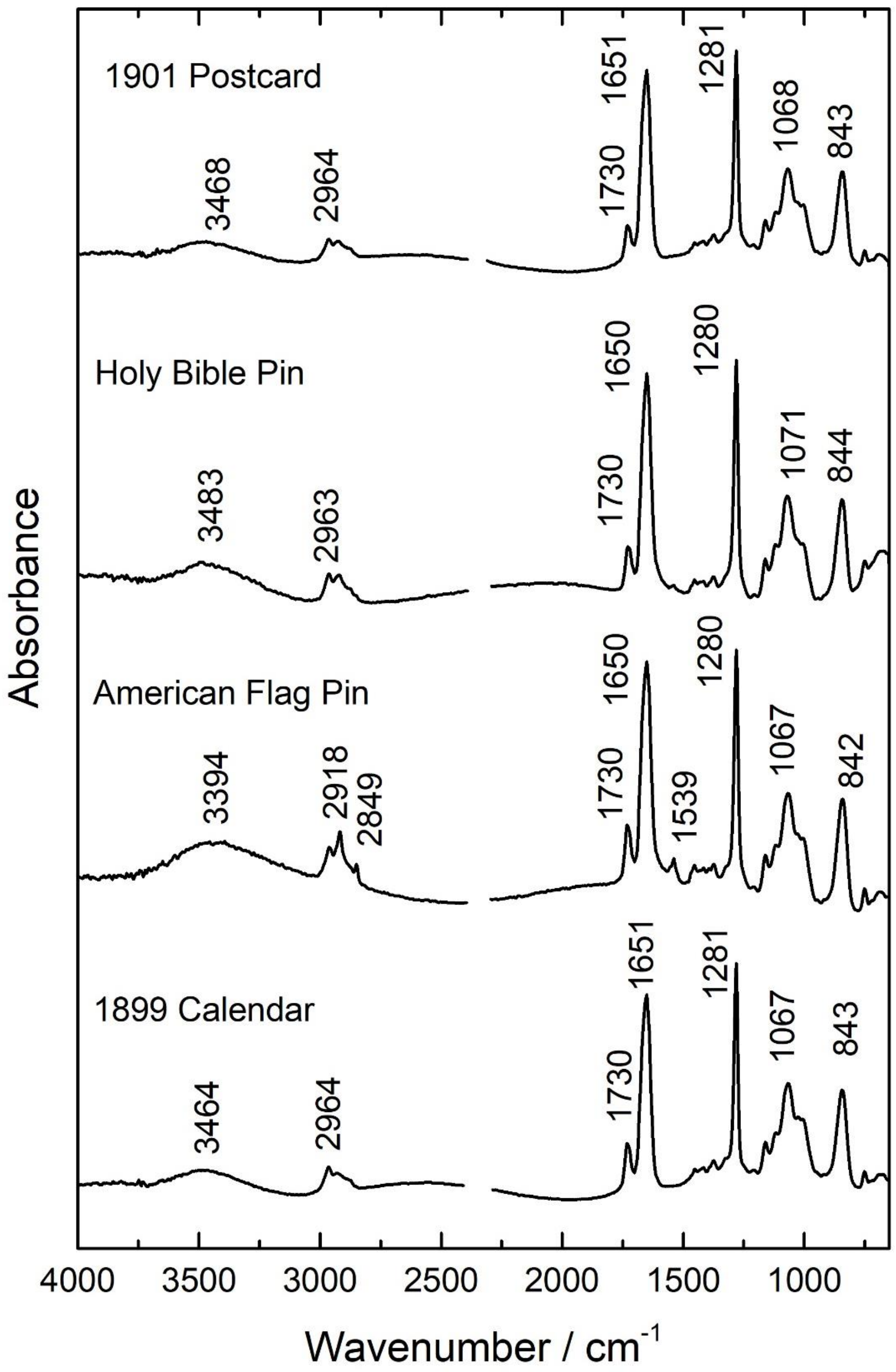

Figure S12. Infrared spectra of Perlov´s celluloid objects analysed. Bands at 2918, 2849 and 1539 cm^{-1} observed in the American flag pin are due to zinc stearate. Using Bussiere et al (2014) calibration curve for the quantification of camphor (A_{1730}/A_{1655} = 0.013 x %camphor, in a rough approximation without considering the degree of substitution), in the calendar we found 18% w/w camphor, in the American flag pin 20%, in the holy bible pin 16% and in the 1901 postcard 15%.

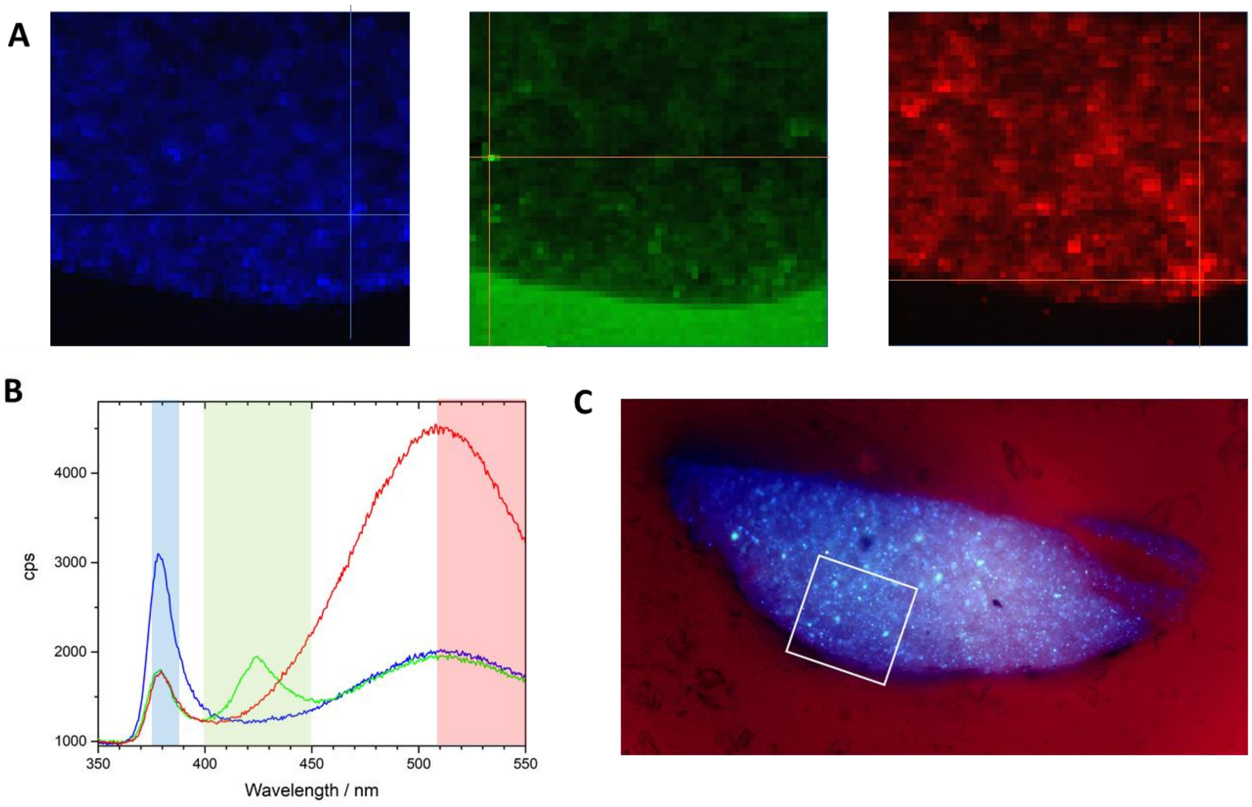

Figure S13. A) POLYPHEME raster scan mapping of the 1901 postcard (3 x 3 μm^2, 5s, 2 accumulation, λ_{exc} = 290 nm). Colors are associated to the following spectral regions: blue for the band edge emission at 385 nm, green for the spectral region between 400-450 nm; red for the region between 510-550 nm (these regions are highlighted in B). **B)** Emission spectra of the pixels marked with a cross in the maps showed in A). **C)** 1901 postcard sample spatially registered false-colour RGB image of the emission at excitations of 365 (blue), 385 (green) and 405 nm (red) with emission bandpass filter 514 nm (30 nm FWHM) The white rectangle marks the POLYPHEME map area.

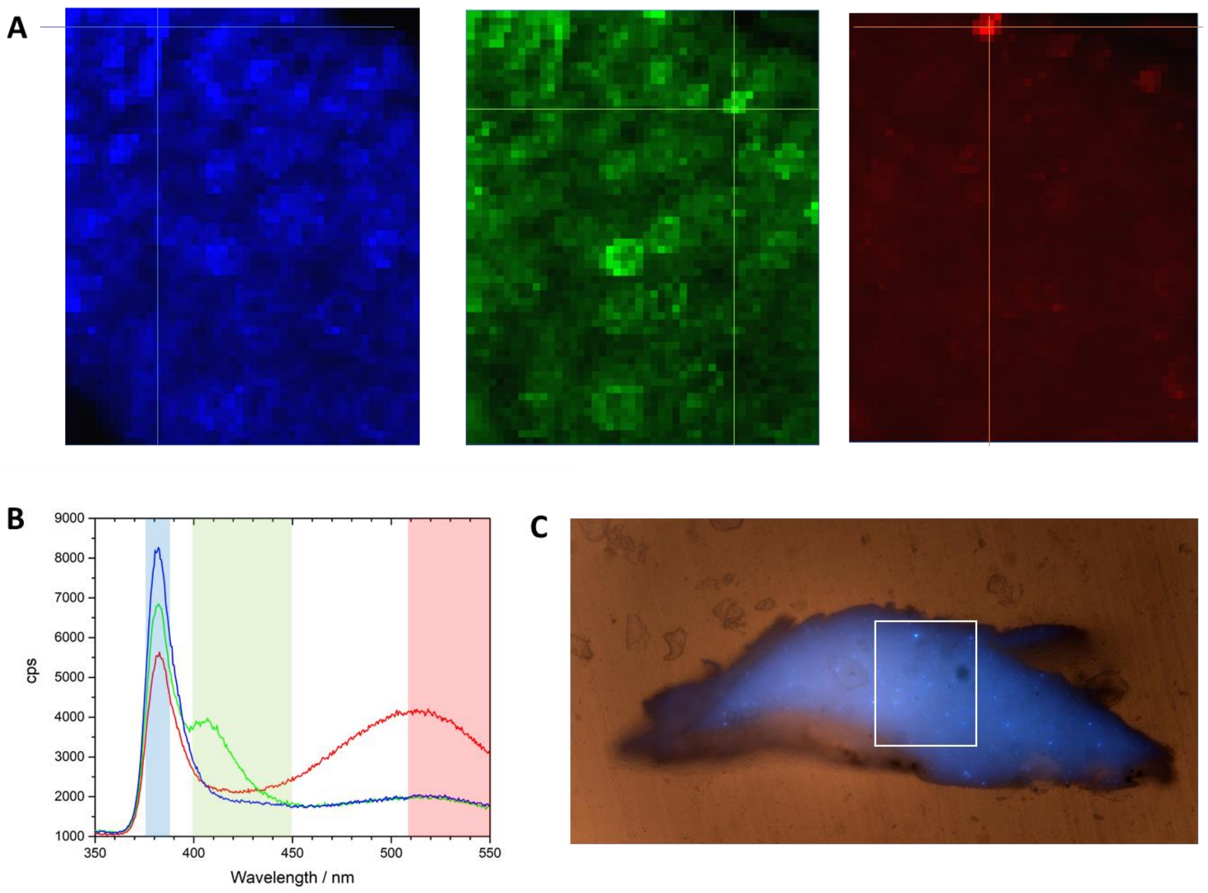

Figure S14. A) POLYPHEME raster scan mapping of the holy bible pin (2 x 2 μm^2, 10s, 1 accumulation, λ_{exc} = 290 nm). Colors are associated to the following spectral regions: blue for the band edge emission at 385 nm, green for the spectral region between 400-450 nm; red for the region between 510-550 nm (these regions are highlighted in B). **B)** Emission spectra of the pixels marked with a cross in the maps showed in A). **C)** Holy bible pin sample spatially registered false-colour RGB image of the emission at excitations of 365 (blue), 385 (green) and 405 nm (red) with emission bandpass filter 514 nm (30 nm FWHM) The white rectangle marks the POLYPHEME map area.

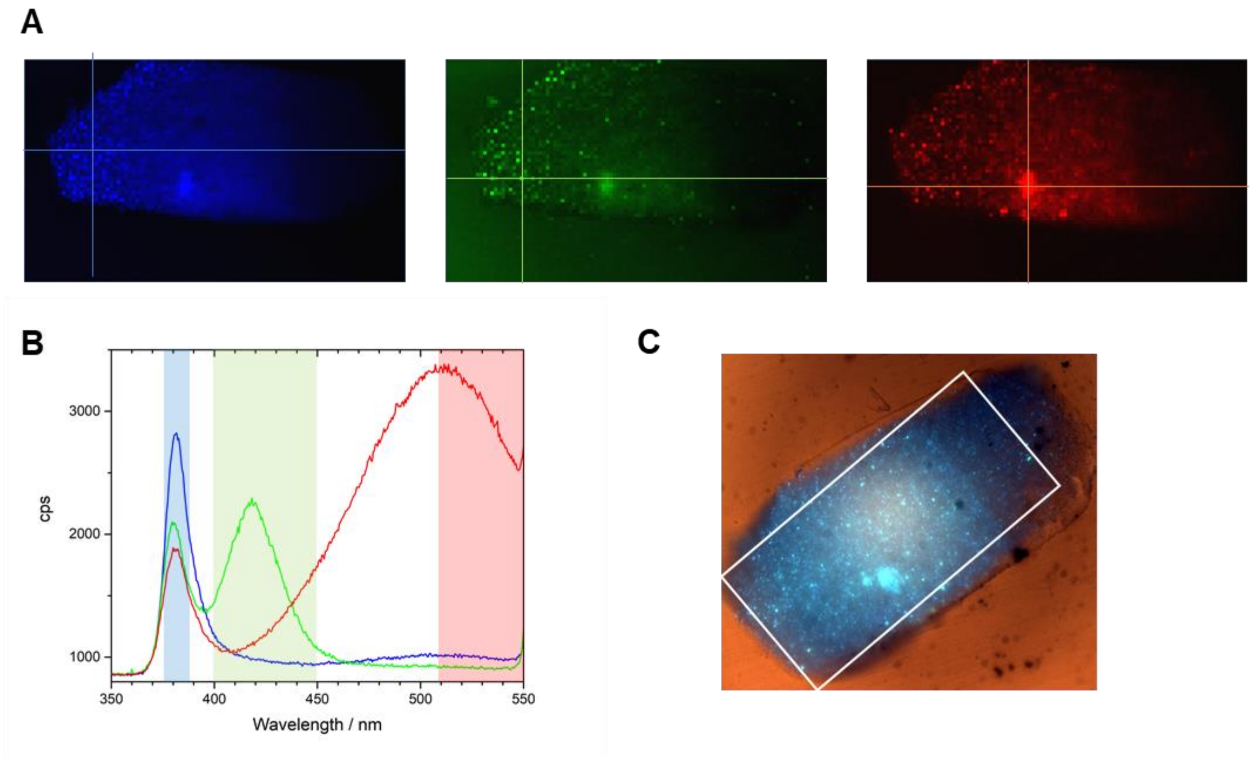

Figure S15. A) POLYPHEME raster scan mapping of the 1899 calendar (6 x 6 μm^2, 2s, 2 accumulations, λ_{exc} = 290 nm). Colors are associated to the following spectral regions: blue for the band edge emission at 385 nm, green for the spectral region between 400-450 nm; red for the region between 510-550 nm (these regions are highlighted in B). **B)** Emission spectra of the pixels marked with a cross in the maps showed in A). **C)** 1899 calendar sample spatially registered false-colour RGB image of the emission at excitations of 365 (blue), 385 (green) and 405 nm (red) with emission bandpass filter 514 nm (30 nm FWHM) The white rectangle marks the POLYPHEME map area.

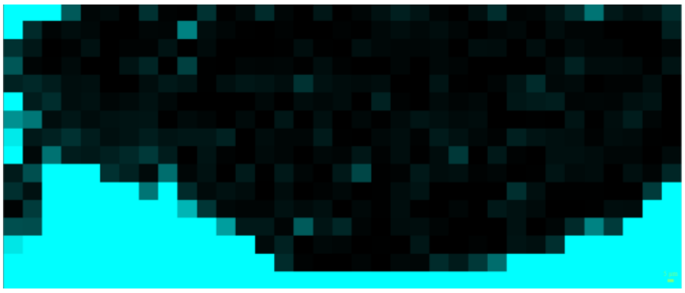

Figure S16. Error map of the DCLS modelling performed to the map of the American flag pin sample. Regions of high errr (bright intensity) indicate a bad fit. Essentially, this map shows the resin surrounding the sample, which does not fit well with the reference component spectra used.

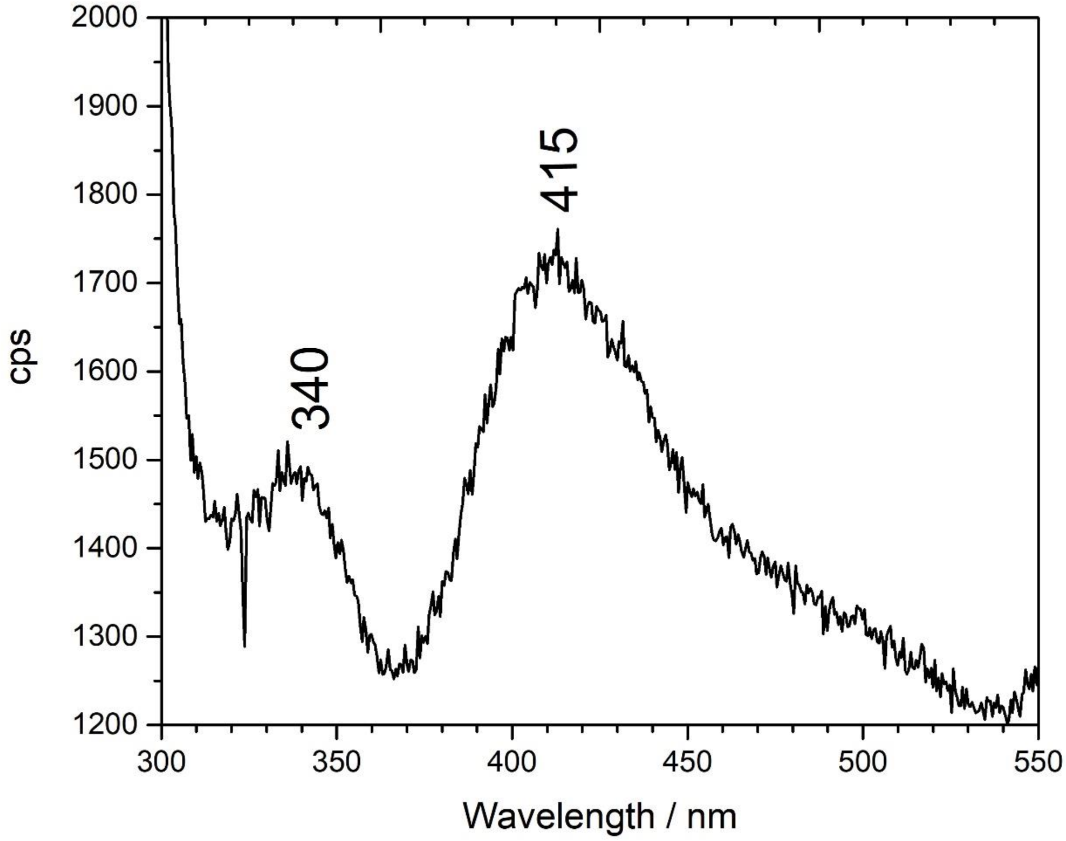

Figure S17. Emission spectrum of the polyester resin used for embedding the celluloid micro samples.
